# Betulinic Acid Attenuates Lipopolysaccharide-Induced Kidney Inflammatory Injury by Suppressing PANoptosis in Weaned Piglets

**DOI:** 10.3390/vetsci13030213

**Published:** 2026-02-25

**Authors:** Yu Yang, Huan Yao, Jiayu He, Zhaoping Ou, You Huang, Wenyu Ba, Ziming Wang, Jiao Wu, Hongyi Ding, Zhuliang Tan, Quanwei Li, Jine Yi, Shuiping Liu

**Affiliations:** 1 Hunan Engineering Research Center of Livestock and Poultry Health Care, College of Veterinary Medicine, Hunan Agricultural University, Changsha 410128, China; 2 SuBait Inc., Dartmouth, NS B2W 6K4, Canada

**Keywords:** betulinic acid, lipopolysaccharide, renal inflammatory injury, PANoptosis, weaned piglets

## Abstract

In the intensive livestock farming industry, weaned piglets are highly prone to renal injury triggered by weaning stress, pathogen infection, and antibiotic abuse. This injury induces metabolic disorders and immunosuppression, severely restricting production efficiency. As a natural pentacyclic triterpene, betulinic acid (BA) exhibits notable biological activities, particularly in anti-inflammatory and antioxidant activities. However, its preventive potential against renal injury in piglets and the underlying mechanisms remain unclear. In this study, BA was administered as a long-term dietary pretreatment prior to lipopolysaccharide (LPS) challenge to evaluate its protective role in a preventive model of renal inflammatory injury in weaned piglets. BA pretreatment significantly mitigated pathological lesions, including renal tubular epithelial cell shedding and interstitial congestion, reduced the renal index, and decreased the concentrations of renal injury markers and serum UREA. In addition, BA pretreatment mitigated the renal oxidative stress and inflammatory injury induced by LPS in piglets. Molecular analyses showed that BA pretreatment was associated with decreased expression of key markers involved in apoptosis, necroptosis, and pyroptosis in renal tissue. Furthermore, protein–protein interaction analysis suggested potential associations between the HMGB1/TLR4/NF-κB signaling pathway and PANoptosis-related processes, providing exploratory and hypothesis-generating support for the proposed regulatory network. Collectively, these findings suggest that dietary BA pretreatment exerts a preventive effect against LPS-induced renal inflammatory injury in weaned piglets, potentially through modulation of HMGB1/TLR4/NF-κB-associated PANoptosis-related pathways, providing a theoretical basis for its application in livestock production.

## Simple Summary

Kidney injury is a common health problem in weaned piglets raised under intensive farming conditions and is often associated with stress, bacterial infection, and inflammation. Such injury can negatively affect animal health and growth performance. Betulinic acid is a natural compound derived from plants and is known for its anti-inflammatory and antioxidant properties. However, its potential role in protecting kidney health in piglets has not been fully explored. In this study, piglets were fed a diet supplemented with betulinic acid for several weeks before being exposed to a bacterial toxin that induces kidney inflammation. The results showed that dietary pretreatment with betulinic acid reduced kidney tissue damage, improved antioxidant capacity, and alleviated inflammatory responses. Changes in several molecular indicators related to cell injury and inflammation were also observed, suggesting that betulinic acid may help regulate multiple biological processes involved in kidney damage. Overall, these findings indicate that long-term dietary supplementation with betulinic acid may help reduce inflammation-related kidney injury in piglets. This study provides useful information for improving animal health management and suggests a potential application of natural plant-derived compounds in livestock production.

## 1. Introduction

Renal injury of weaned piglets is a common pathological condition in intensive swine production, closely associated with multiple factors, including weaning stress, nutritional imbalance, pathogenic microbial infections, and antibiotic misuse. Specifically, weaning stress can lead to intestinal dysbiosis, immunosuppression, and elevated cortisol concentrations. This stress state can reduce the glomerular filtration rate and impair the renal tubular reabsorption function, which in turn triggers renal tubular epithelial cell damage, interstitial fibrosis, and renal insufficiency [1,2]. Furthermore, certain pathogens could activate Toll-like receptors (TLRs) signaling pathways, resulting in elevated pro-inflammatory cytokine production and inducing oxidative stress, ultimately causing renal injury [3]. Current strategies for managing renal injury in piglets rely heavily on antibiotic and symptomatic treatments, which are constrained by drug resistance, inconsistent efficacy, and increasing concerns regarding nephrotoxicity. In addition to affecting production performance, renal inflammatory injury in weaned piglets represents a clinically relevant condition in veterinary medicine. Elucidating its underlying mechanisms may therefore contribute to a broader understanding of renal inflammatory disorders in domestic animals.

In recent years, natural plant extracts have gained significant research interest due to their multi-targeting potential and bioactive properties. Betulinic acid (BA) is a naturally occurring pentacyclic triterpenoid obtained from the bark of white birch and is known for its multiple bioactivities, such as anti-inflammatory, anti-apoptotic, and antioxidant effects [4,5]. Previous studies have shown that BA attenuated spinal cord injury-induced neuroinflammation and zearalenone-induced liver inflammation in the mouse model [6,7]. Additionally, BA could upregulate the concentrations of redox-related proteins to reduce oxidative damage, which has a neuroprotective effect on early brain injury [8]. In this context, oxidative stress-related parameters, such as lipid peroxidation products and antioxidant enzyme activities, are often interpreted as integrative indicators of systemic physiological disturbance rather than isolated pathway-specific readouts. In veterinary large animal models, these biomarkers have been widely applied to reflect the combined impact of inflammation, metabolic stress, and tissue injury [9]. Collectively, these findings suggest that BA exhibits organ-protective potential across multiple experimental settings, primarily associated with its anti-inflammatory and antioxidant properties. Although BA has been reported to exert renoprotective effects in certain experimental models, its role in alleviating kidney injury in weaned piglets remains insufficiently explored, particularly under post-weaning stress conditions relevant to intensive swine production.

Emerging evidence indicated that the high-mobility group box 1 (HMGB1)/TLR4/nuclear factor-kappa B (NF-κB) axis may act as an upstream regulatory hub in the development of kidney damage and has been implicated in crosstalk with PANoptosis, an emerging integrative concept of programmed cell death encompassing necroptosis, apoptosis, and pyroptosis [10,11]. Upon release from stressed or damaged renal tubular cells, HMGB1 functions as a danger-associated molecular pattern that engages TLR4 on immune cells, thereby triggering downstream NF-κB pathway activation [12]. Under TNF-α stimulation, NF-κB activation enhances cellular susceptibility to necroptosis via the receptor-interacting protein kinase 1 (RIPK1)/RIPK3/mixed lineage kinase domain-like protein (MLKL) axis [13]. Furthermore, necroptosis-induced HMGB1 release may further potentiate TLR4/NF-κB signaling, establishing a self-sustaining feed-forward inflammatory loop [14,15]. Beyond its regulation of necroptosis, the HMGB1/TLR4/NF-κB-driven inflammatory milieu amplifies reactive oxygen species (ROS)-dependent oxidative stress alongside mitochondrial impairment [16]. This subsequently activates cytochrome-C-mediated apoptosis and NLR family, pyrin domain-containing protein 3 (NLRP3) inflammasome-driven pyroptosis, thereby jointly constituting the PANoptosis program [17,18]. Therefore, modulation of the HMGB1/TLR4/NF-κB signaling axis and its potential association with PANoptosis-related pathways may represent a promising strategy for mitigating renal inflammatory injury. However, it is still unknown whether BA could alleviate lipopolysaccharide (LPS)-induced kidney damage in piglets through regulation of the HMGB1/TLR4/NF-κB signaling pathway.

In this study, a weaned piglet model of LPS-induced renal injury was used to systematically examine the effects of BA on renal morphology and function, oxidative stress, inflammatory responses, and distinct renal cell death modalities. Additionally, this study aimed to clarify whether the renoprotective effects of BA are associated with PANoptosis mediated by the HMGB1/TLR4/NF-κB signaling pathway. The present study aimed to establish a theoretical basis for the use of natural plant extracts in protecting renal function from injury in livestock.

## 2. Materials and Methods

### 2.1. Establishment of Experimental Model

All experiments were conducted following the protocol approved by the Animal Care Committee of Hunan Agricultural University (Approval No. Lunshen 2022-31, approved on 11 March 2022) and the relevant animal welfare regulations of the University. Male Duroc × Landrace × Yorkshire (DLY) piglets were weaned at 28 days of age and randomly distributed into four treatment groups (*n* = 8): Control, LPS, BA, and BA + LPS groups. A basal diet meeting the NRC (2012) recommendations was provided to all animals, with the corresponding nutrient composition listed in Table S1. To establish a preventive model of renal injury, piglets in the BA and BA + LPS groups received dietary supplementation with BA (60 mg/kg diet; purity ≥ 98%; Xi’an Beijino Biotechnology Co., Ltd., Xi’an, China) for 28 consecutive days prior to LPS challenge, following a 5-day acclimatization period. Piglets in the Control and LPS groups were fed the basal diet throughout the experimental period. To establish a sustained inflammatory challenge that mimics repeated endotoxin exposure during the post-weaning period, LPS derived from Escherichia coli O55:B5 (L2880, Sigma-Aldrich, St. Louis, MO, USA) was administered intraperitoneally at 10 μg/kg BW on experimental days 21 and 28 to piglets in the LPS and BA + LPS groups, while piglets in the Control and BA groups received sterile physiological saline at the corresponding time points. The BA and LPS doses were selected based on previous experimental studies and published literature demonstrating their efficacy and safety in pig models [19]. At 4 h following LPS administration on day 28th, blood was obtained using the anterior vena cava as the sampling site. After blood collection, piglets were anesthetized with 80 mg/kg BW sodium pentobarbital. Upon reaching complete anesthesia, the piglets were humanely euthanized. Kidney tissues were promptly excised, rinsed thoroughly with ice-cold normal saline, and subsequently stored at −80 °C for subsequent experimental analyses.

### 2.2. Histopathological Observation of Kidney

Kidneys fixed with 4% paraformaldehyde were paraffin-embedded, sliced into 4–6 μm sections, and routinely stained with hematoxylin–eosin for histological evaluation.

### 2.3. Serum Biochemical Indicators

Serum CREA and UREA concentrations were quantified using an automated biochemistry (Meryer Chemicals, Shanghai, China) analyzer with commercial kits (Meryer Chemicals, Shanghai, China).

### 2.4. Primer Design and Real-Time Quantitative PCR Analysis (qRT-PCR)

Total RNA from kidney tissues was purified using AG RNAex Pro kit (Accurate Biotechnology, Changsha, China) and subsequently subjected to cDNA synthesis with the Evo M-MLV RT reagent kit (Accurate Biotechnology, Changsha, China) following quality validation. The reaction system prepared by the SYBR Green I kit (Accurate Biotechnology, Changsha, China) was amplified on a real-time fluorescence quantitative PCR instrument and detected by fluorescence quantitative PCR. Relative mRNA expression was calculated using GAPDH as the internal reference, with the 2^−ΔΔCt^ approach, and primer sequences (Shanghai Shenggong Biotechnology Co., Ltd., Shanghai, China) were presented in Table S2.

### 2.5. Antioxidant Capacity of Kidneys

Following homogenization, renal samples were centrifuged at 12,000× *g* for 15 min at 4 °C, after which the supernatant fractions were harvested. Commercial assay kits were used to determine the concentrations of malondialdehyde (MDA), total antioxidant capacity (T-AOC), superoxide dismutase (SOD) activity, and glutathione (GSH) activity according to the manufacturer’s instructions (Nanjing Jiancheng Bioengineering Institute, China).

### 2.6. ELISA Detection

Serum KIM-1 concentrations were determined with a porcine-specific ELISA kit (Jiangsu Jingmei Biotechnology Co., Ltd., Yancheng, China) in accordance with the manufacturer’s instructions, with optical density read at 450 nm.

### 2.7. Western Blot Analysis

Kidney protein was isolated with RIPA lysis buffer (Servicebio, Wuhan, China), and then the total protein concentration was measured using the BCA kit (Heruibio, Fujian, China). Separated proteins (8–12% SDS-PAGE) were transferred to PVDF membranes, blocked with 5% milk, and incubated with primary antibodies at 4 °C overnight and the secondary antibody (ABclonal, Wuhan, China) for 45 min at room temperature. The highly sensitive ECL chemiluminescence reagent (NCM Biotech, Suzhou, China) was dropped onto the membrane, and then the image was obtained in the digital chemiluminescence imaging system. The primary protein antibodies are shown in Table S3.

### 2.8. Immunofluorescence Analysis

Paraffin sections were processed through deparaffinization and rehydration, followed by antigen retrieval, and then exposed to primary antibodies at 4 °C overnight. Cy3-conjugated secondary antibodies were used to visualize target protein localization within renal tissue sections. After treatment with Cy3-labeled secondary antibodies (Servicebio, Wuhan, China), nuclei were counterstained with DAPI, and fluorescence was observed by microscopy. The primary protein antibodies are shown in Table S3.

### 2.9. Immunohistochemical Analysis of KIM-1

Paraffin sections underwent deparaffinization, rehydration, and antigen retrieval, and then were incubated with anti-KIM-1 antibody (Abmart, Shanghai, China) overnight at 4 °C and HRP-conjugated secondary antibodies (Servicebio, Wuhan, China). Immunoreactivity was visualized using DAB with hematoxylin counterstaining, and a light microscope was used to acquire the images.

### 2.10. TUNEL Analysis

Apoptotic cells were detected by TUNEL staining with a FITC-conjugated detection kit (Vazyme, Nanjing, China) according to the manufacturer’s instructions, and images were captured under a fluorescence microscope.

### 2.11. Protein–Protein Interaction (PPI) Network Analysis

The interaction network linking PANoptosis-related proteins with components of the HMGB1/TLR4/NF-κB signaling pathway was constructed using the STRING database, which incorporates both experimentally validated and computationally predicted associations. For species available in the database, the PPI network was generated by inputting the list of target genes.

### 2.12. Statistical Analysis

Statistical analyses were performed using SPSS version 27.0. Data are presented as mean ± SEM. Prior to statistical analysis, data normality and homogeneity of variances were assessed using the Shapiro–Wilk test and Levene’s test, respectively. Differences among multiple groups were evaluated by one-way analysis of variance (ANOVA), followed by the Student–Newma–Keuls (SNK) post hoc test. Statistical significance was defined as *p* < 0.05 or *p* < 0.01.

## 3. Results

### 3.1. BA Pretreatment Attenuated LPS-Induced Renal Injury in Piglets

**Figure 1:**
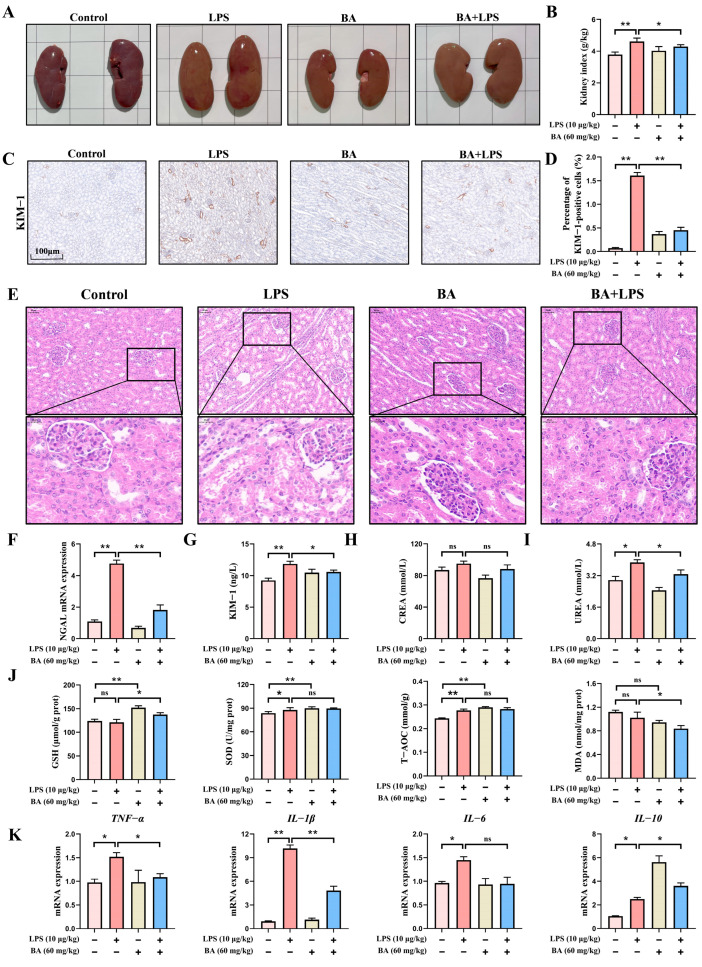
Effect of BA pretreatment on LPS-induced renal injury in piglets. (**A**) Morphological changes in renal tissue. (**B**) Renal index. (**C**) Immunohistochemical staining revealed the distribution of KIM-1 protein (scale bar: 100 μm). (**D**) Quantitative densitometric evaluation of KIM-1 protein concentrations. (**E**) Representative H&E-stained kidney sections from Control, LPS, BA, and BA + LPS groups (scale bar: 50 μm and 20 μm). (**F**) qRT-PCR-based assessment of *NGAL* mRNA expression in the kidney. (**G**) The serum level of KIM-1 was quantified by ELISA. (**H**,**I**) The serum concentrations of CERA and UERA. (**J**) The concentrations of GSH, SOD, T-AOC, and MDA in the kidneys of piglets. (**K**) qRT-PCR-based assessment of *TNF-α*, *IL-1β*, *IL-6*, and *IL-10* mRNA expression in the kidney. Statistical analyses are reported as mean values ± SEM, with significance assigned at probability levels of *p* < 0.05 (*) and *p* < 0.01 (**) compared between the two groups, while “ns” indicates no statistically significant difference.

As illustrated in Figure 1A,B, BA pretreatment markedly alleviated LPS-induced renal enlargement and congestion and resulted in a lower kidney-to-body weight ratio. H&E staining revealed that BA pretreatment alleviated LPS-induced renal histopathological alterations in piglets, as evidenced by a more preserved tubular architecture, reduced tubular epithelial cell swelling and vacuolar degeneration, and less prominent tubular luminal dilation, accompanied by relatively limited interstitial inflammatory cell infiltration (Figure 1E). Immunohistochemical analysis revealed that KIM-1 protein was primarily detected in renal tubular epithelial cells, whereas glomerular structures exhibited minimal to no staining (Figure 1C). The positive signals were mainly localized to the apical membrane and cytoplasm of tubular epithelial cells, indicating tubular involvement following LPS challenge. Notably, BA pretreatment markedly reduced KIM-1 protein distribution in renal tubules, accompanied by decreased serum KIM-1 concentrations and reduced *NGAL* mRNA expression compared with the LPS group (Figure 1C,D,F,G). Serum biochemical analysis showed that BA pretreatment significantly reduced serum UREA concentrations compared with the LPS group, whereas no significant change in serum CREA concentrations was observed between the BA + LPS and LPS groups (Figure 1H,I). Compared to the LPS group, renal GSH concentrations were significantly elevated, whereas renal MDA concentration was modestly decreased in piglets in the BA + LPS group (Figure 1J). As depicted in Figure 1K, LPS exposure resulted in a marked upregulation of *TNF-α*, *IL-1β*, and *IL-6* mRNA levels in piglet kidneys. In comparison with the LPS group, administration of BA markedly suppressed the mRNA expression of pro-inflammatory cytokines (*TNF-α*, *IL-1β*, and *IL-6*) while enhancing *IL-10* mRNA expression. Collectively, these findings supported a protective role of dietary BA against LPS-induced oxidative and inflammatory damage in the kidneys of piglets.

### 3.2. BA Pretreatment Attenuated LPS-Induced Renal Apoptosis in Piglets

**Figure 2:**
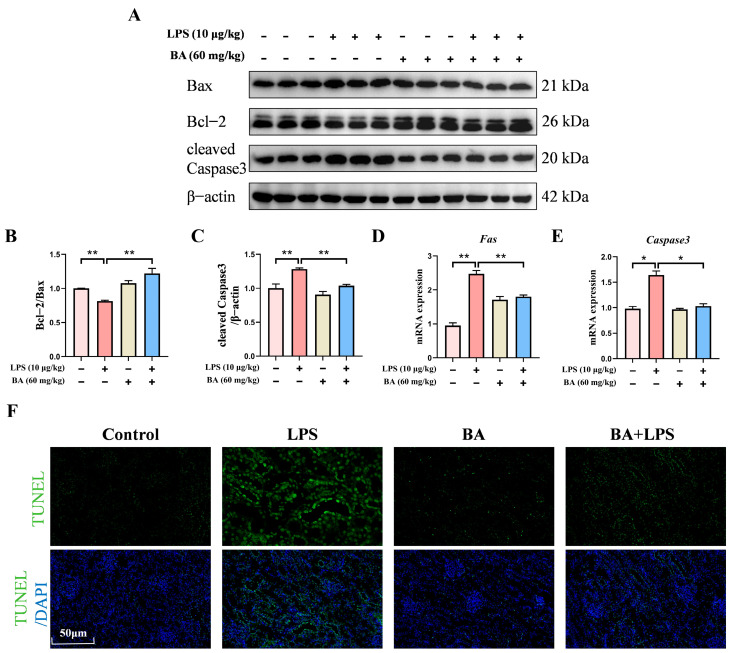
Effect of BA pretreatment on LPS-induced renal apoptosis in piglets. (**A**) Western blot detection of Bcl-2, Bax, and cleaved Caspase-3 proteins in the kidney. (**B**,**C**) Quantification of the protein band density for Bcl-2/Bax ratios and cleaved Caspase 3 in the kidney. (**D**,**E**) qRT-PCR-based assessment of *FAS* and *Caspase3* mRNA expression in the kidney. (**F**) The distribution of apoptotic cells was assessed by TUNEL staining (scale bar: 50 μm). Statistical analyses are reported as mean values ± SEM, with significance assigned at probability levels of *p* < 0.05 (*) and *p* < 0.01 (**) compared between the two groups, while “ns” indicates no statistically significant difference.

To clarify the contribution of BA to renal apoptosis under LPS treatment, apoptosis-related markers were analyzed. It was observed that BA pretreatment markedly increased the Bcl-2/Bax protein ratio while suppressing the expression of cleaved Caspase 3 (Figure 2A–C). qRT-PCR results revealed that BA pretreatment significantly downregulated the mRNA expression of *FAS* and *Caspase 3* compared to the LPS group (Figure 2D,E). In addition, the number of renal TUNEL-positive cells was lower in BA-treated piglets after LPS exposure (Figure 2F). Overall, these results confirmed that BA supplementation could mitigate LPS-induced renal apoptosis in piglets.

### 3.3. BA Pretreatment Attenuated LPS-Induced Necroptosis in the Kidney of Piglets

**Figure 3:**
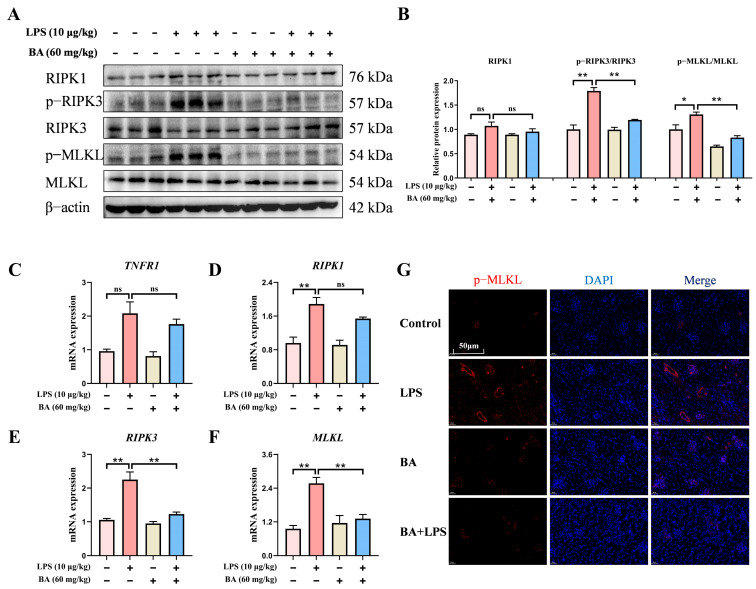
Effect of BA pretreatment on LPS-induced renal necroptosis in piglets. (**A**) Western blot analysis of RIPK1, p-RIPK3, RIPK3, p-MLKL, and MLKL proteins in the kidney. (**B**) Quantification of the protein band density for RIPK1, p-RIPK3/RIPK3, and p-MLKL/MLKL in the kidney. (**C**–**F**) qRT-PCR-based assessment of *TNFR1*, *RIPK1*, *RIPK3*, and *MLKL* mRNA expression in the kidney. (**G**) Immunofluorescence analysis showed the distribution of p-MLKL (scale bar: 50 μm). Statistical analyses are reported as mean values ± SEM, with significance assigned at probability levels of *p* < 0.05 (*) and *p* < 0.01 (**) compared between the two groups, while “ns” indicates no statistically significant difference.

To further evaluate the impact of BA on LPS-induced renal necroptosis in piglets, the key necroptosis-associated mRNA and proteins were evaluated. In comparison with the Control group, LPS exposure significantly activated the phosphorylation levels of RIPK3 and MLKL proteins, while also upregulating the RIPK1 protein expression. Compared to the LPS group, BA administration markedly decreased the phosphorylated forms of RIPK3 and MLKL (Figure 3A,B). Consistently, qRT-PCR results further confirmed that BA pretreatment led to a pronounced downregulation of *RIPK1*, *RIPK3*, and *MLKL* at the mRNA level (Figure 3C–F). Immunofluorescence staining demonstrated a reduced red fluorescence signal of p-MLKL in the BA pretreatment group (Figure 3G). In conclusion, these observations implied that BA supplementation attenuated LPS-induced renal necroptosis in piglets.

### 3.4. BA Pretreatment Attenuated LPS-Induced Pyroptosis in the Kidney of Piglets

**Figure 4:**
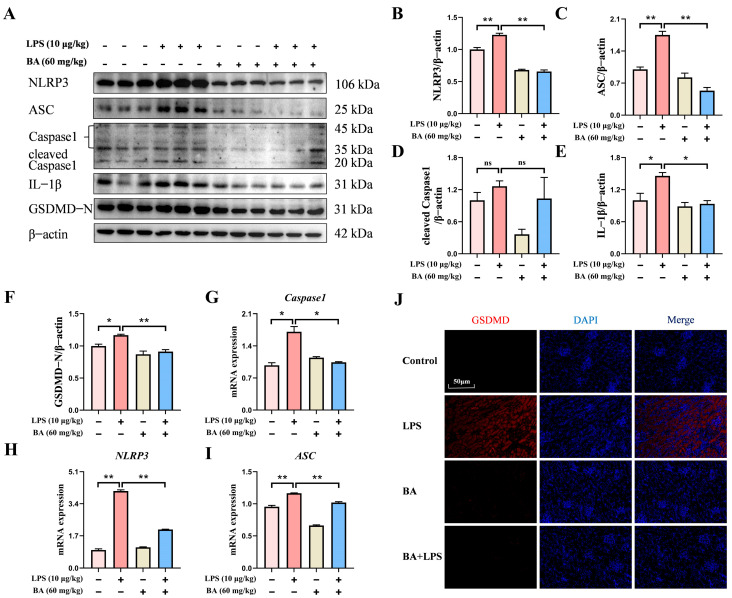
Effect of BA pretreatment on LPS-induced renal pyroptosis in piglets. (**A**) Western blot analysis of pyroptosis-related proteins in the kidney (NLRP3, ASC, cleaved Caspase 1, IL-1β, and GSDMD-N). (**B**–**F**) Quantification of protein band densities for pyroptosis-related proteins in the kidney. (**G**–**I**) qRT-PCR-based assessment of *Caspase1*, *NLRP3*, and *ASC* mRNA expression in the kidney. (**J**) Immunofluorescence analysis showed the distribution of GSDMD (scale bar: 50 μm). Statistical analyses are reported as mean values ± SEM, with significance assigned at probability levels of *p* < 0.05 (*) and *p* < 0.01 (**) compared between the two groups, while “ns” indicates no statistically significant difference.

To further evaluate BA involvement in LPS-triggered pyroptosis, the abundance of key pyroptosis-related proteins was determined by Western blotting. As shown in Figure 4A–F, the protein levels of NLRP3, ASC, cleaved caspase-1, GSDMD, and IL-1β were substantially lower in the BA-treated piglets following LPS challenge than in those exposed to LPS alone. Meanwhile, BA pre-treatment significantly diminished the mRNA expression of *NLRP3*, *ASC*, and *Caspase-1* compared with the LPS group (Figure 4G–I). Immunofluorescence analysis revealed a reduced fluorescence intensity of GSDMD in the BA + LPS group compared with LPS exposure alone (Figure 4J). Collectively, these results suggested that BA supplementation alleviated LPS-triggered renal pyroptosis in piglets.

### 3.5. BA Pretreatment Suppressed LPS-Induced Renal Inflammation Through HMGB1/TLR4/NF-κB Signaling Pathway in Piglets

**Figure 5:**
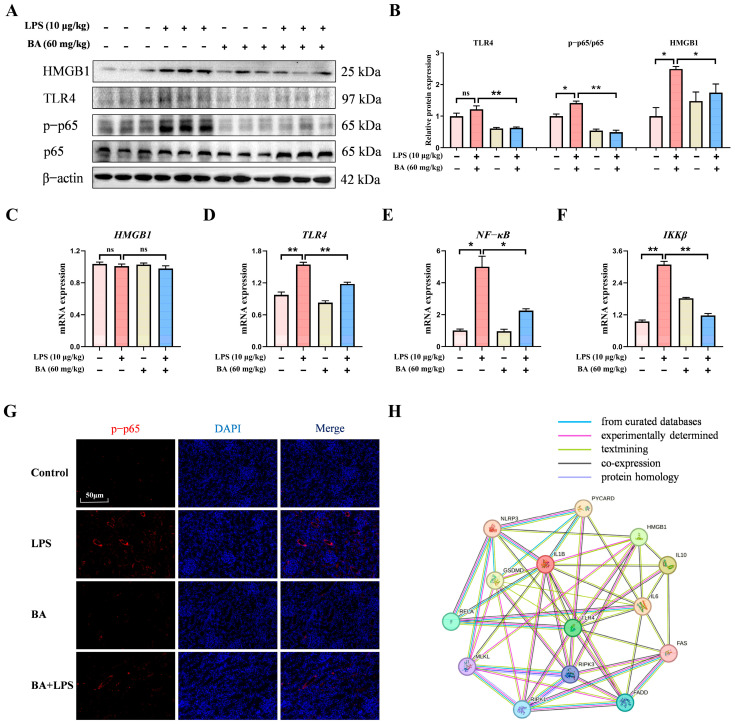
Effect of BA on LPS-induced inflammatory response and HMGB1/TLR4/NF-κB signaling pathways in the kidney of piglets. (**A**) Western blot detection of HMGB1, TLR4, p65, and p-p65 proteins in the kidney. (**B**) Quantification of protein band densities for TLR4, p-p65/p65 ratios, and HMGB1 in the kidney. (**C**–**F**) qRT-PCR-based assessment of *HMGB1*, *TLR4*, *NF-κB*, and *IKKβ* mRNA expression in the kidney. (**G**) Immunofluorescence analysis showed the distribution of p-p65 (scale bar: 50 μm). (**H**) Protein interaction network depicting relationships among HMGB1/TLR4/NF-κB pathway components and necroptosis, apoptosis, pyroptosis markers, and inflammatory cytokines in the kidney. Statistical analyses are reported as mean values ± SEM, with significance assigned at probability levels of *p* < 0.05 (*) and *p* < 0.01 (**) compared between the two groups, while “ns” indicates no statistically significant difference.

To elucidate the regulatory function of BA pretreatment in LPS-induced renal inflammation, key components of the HMGB1/TLR4/NF-κB signaling cascade were subsequently analyzed. Western blot results indicated that BA pretreatment markedly decreased the HMGB1 and TLR4 protein expression and reduced the p-p65/p65 ratio following LPS stimulation (Figure 5A,B). qRT-PCR results revealed that BA pretreatment significantly downregulated the *TLR4*, *NF-κB*, and *IKKβ* mRNA levels compared with the LPS group (Figure 5C,F). Additionally, immunofluorescence assay results showed that the fluorescence intensity of p-p65 protein was obviously decreased following BA pretreatment under LPS stimulation (Figure 5G). Furthermore, PPI analysis suggested potential functional associations between the HMGB1/TLR4/NF-κB signaling pathway and markers related to necroptosis, apoptosis, pyroptosis, and inflammatory responses, providing exploratory and hypothesis-generating evidence (Figure 5H). Collectively, these findings suggested that BA supplementation attenuated LPS-induced renal inflammation in piglets by suppressing the HMGB1/TLR4/NF-κB signaling pathway.

## 4. Discussion

Owing to incomplete physiological development and an immature immune status in weaned piglets, they are particularly susceptible to renal injury induced by pathogenic microbial infections, antibiotic overuse, or oxidative stress, with characteristic manifestations including anorexia, growth retardation, diarrhea, dehydration, and azotemia [20]. To model renal injury during the weaning transition, a clinically relevant LPS-induced sepsis model was established by intraperitoneal injection of LPS at 10 μg/kg BW [21]. LPS exposure elicited evident renal histopathological alterations, characterized by tubular epithelial cell swelling and partial exfoliation, luminal dilation, and mild interstitial inflammatory cell infiltration [22], confirming successful model establishment.

BA, a plant-derived pentacyclic triterpenoid, has been reported to exert anti-inflammatory and antioxidant effects in various disease models, including chronic kidney disease [23]. KIM-1 is widely recognized as a sensitive and early biomarker of renal tubular injury, and its detection provides important histological and biochemical evidence for evaluating the severity of tubular damage. In the present study, BA pretreatment reduced serum KIM-1 concentrations and NGAL mRNA expression, indicating an attenuation of renal tubular injury. Although BA partially decreased serum UREA concentrations, no significant change in CREA was observed, likely reflecting early-stage tubular injury with relatively preserved glomerular filtration. This pattern is consistent with prerenal or early AKI features, where UREA elevation precedes CREA changes [24], which may further explain our observed results. In summary, BA has shown potential as a feed additive for mitigating renal tubular epithelial cell injury in piglets under LPS challenge.

Oxidative stress and inflammation are closely interconnected processes that jointly contribute to renal injury [25,26]. Bioactive compounds have been shown to mitigate kidney damage by enhancing antioxidant defenses and suppressing inflammatory signaling [27,28,29]. BA is increasingly recognized for its therapeutic potential based on its antioxidative and anti-inflammatory activities. In models of renal disease, BA has been shown to activate Nrf2 signaling, reduce ROS accumulation, and alleviate inflammatory injury [30]. Moreover, BA effectively exhibited protective effects against inflammation-induced tissue damage, including cyclophosphamide (CYP)-induced intestinal mucosal injury [31], further supporting its regulatory role in oxidative–inflammatory pathways. In the present study, BA pretreatment significantly increased renal GSH and significantly decreased MDA, whereas SOD and T-AOC showed no significant changes, indicating that BA improved redox status mainly through reinforcing the glutathione system and limiting lipid peroxidation. In addition, BA alone elevated renal GSH, SOD, and T-AOC without significantly affecting MDA, consistent with previous reports that BA enhances antioxidant capacity and confers renal protection [32,33,34]. Notably, LPS transiently upregulated the activities of SOD and T-AOC, which likely reflected the adaptive redox response of weaned piglets [35]. Meanwhile, BA pretreatment markedly suppressed the mRNA levels of pro-inflammatory cytokines (*TNF-α*, *IL-1β*, and *IL-6*), while promoting the *IL-10* mRNA level during renal inflammatory responses triggered by LPS. Collectively, these observations suggested that BA alleviated renal damage in piglets by concurrently strengthening antioxidant defenses and inhibiting inflammatory responses.

The kidney is a sentinel organ highly susceptible to LPS-induced injury, in which apoptosis, necroptosis, and pyroptosis are concurrently involved in the amplification of tissue damage and inflammatory responses, collectively contributing to organ dysfunction [36,37,38,39]. Recent studies have proposed the concept of PANoptosis as an integrated form of inflammatory cell death characterized by extensive crosstalk among multiple programmed cell death pathways and shared molecular regulators. Dysregulation of this network triggers a vicious positive feedback loop termed “cell death–inflammation” [11]. Meanwhile, BA has been reported to provide protection via the concurrent inhibition of apoptosis and pyroptosis across multiple targets [40]. For example, BA has been shown to attenuate CYP-triggered hepatic apoptosis by blocking the oxidative stress-activated mitochondrial apoptotic pathway [41]. In addition, in spinal cord injury models, BA limited pyroptosis by enhancing autophagic activity through AMPK-mTOR-TFEB signaling [42]. Nevertheless, whether BA influences necroptosis pathways has yet to be clarified. In the present study, BA pretreatment was associated with coordinated regulation of key molecular markers involved in apoptosis, necroptosis, and pyroptosis. Specifically, BA pretreatment was associated with an increased Bcl-2/Bax ratio and reduced Fas and Caspase-3 expression, indicating attenuation of apoptosis-related signaling. However, these molecular changes represent indirect indicators rather than direct functional evidence of apoptosis [43]. BA pretreatment also targeted necroptosis by downregulating the RIPK1/RIPK3/MLKL axis and inhibiting p-MLKL membrane translocation, thereby disrupting DAMP release and inflammatory amplification. Furthermore, BA pretreatment suppressed pyroptosis via limiting NLRP3 inflammasome activity and its downstream effector GSDMD, leading to reduced IL-1β production and pore-driven cell lysis. Collectively, these findings suggest that BA alleviated LPS-induced renal inflammatory injury in piglets through coordinated modulation of PANoptosis-related pathways.

HMGB1 has been widely recognized as a key pro-inflammatory mediator that activates the TLR4 signaling pathway, triggering the release of downstream cytokines and promoting PANoptosis-related cell death, thereby amplifying inflammatory injury in multiple tissues [44,45,46,47]. Recent evidence further indicated that BA could alleviate inflammatory injury across multiple disease models by inhibiting the TLR4/NF-κB signaling pathway [48,49]. In the present study, BA pretreatment was observed to significantly suppress the release of HMGB1, suggesting its potential to interfere with the HMGB1/TLR4 signaling pathway. Based on these observations, BA was considered to be potentially involved in the regulation of LPS-triggered renal injury via the HMGB1/TLR4/NF-κB signaling axis. Therefore, PPI network analysis was conducted as an exploratory approach to examine potential molecular associations. Our results revealed that BA targets multiple nodes associated with HMGB1, TLR4, NF-κB, and PANoptosis-related molecules, indicating extensive regulatory crosstalk. Consistently, Western blot and qRT-PCR results revealed that BA pretreatment significantly suppressed the mRNA and protein levels of molecules associated with the TLR4/NF-κB signaling pathway in the kidneys of piglets challenged with LPS.

Several limitations should be acknowledged. In the present study, the integration of apoptosis, necroptosis, and pyroptosis under the framework of PANoptosis is conceptual and based on coordinated changes in related molecular markers rather than direct experimental demonstration of a unified PANoptotic process. The conclusions are primarily derived from gene and protein expression data, which provide indirect and associative evidence. Moreover, although BA pretreatment consistently suppressed HMGB1/TLR4/NF-κB signaling and multiple PANoptosis-associated markers, the current findings support a regulatory association rather than definitive upstream causality. Confirmation of direct mechanistic links would require intervention-based approaches, such as pharmacological inhibition or genetic manipulation of key signaling components. Therefore, the present results should be interpreted as indicative of coordinated modulation of inflammatory and cell death pathways, and further functional validation is warranted to clarify the precise mechanistic relationships involved.

## 5. Conclusions

Our study demonstrated that long-term dietary pretreatment with BA was associated with alleviation of LPS-induced renal inflammatory injury and oxidative stress in weaned piglets. These protective effects were accompanied by coordinated modulation of HMGB1/TLR4/NF-κB signaling and PANoptosis-related molecular markers. This study provides new insight into the potential application of BA as a preventive nutritional strategy for maintaining renal health in livestock production.

## Data Availability

The original contributions presented in this study are included in the article and Supplementary Materials. Further inquiries can be directed to the corresponding author.

## Supplementary Materials

The following supporting information can be downloaded at: https://www.mdpi.com/article/10.3390/vetsci13030213/s1, Table S1: Feed nutrient composition and inclusion levels; Table S2: primer sequences applied for qRT-PCR analysis; Table S3: the antibody information used for Western blot in piglets.
