# Supplementary material for: Betulinic Acid Attenuates Lipopolysaccharide-Induced Kidney Inflammatory Injury by Suppressing PANoptosis in Weaned Piglets
Source: Vet Sci. 2026 Feb 25;13(3):213. doi: 10.3390/vetsci13030213 (PMC13030141; doi:10.3390/vetsci13030213)
Supplement: Supplementary file 1 [file vetsci-13-00213-s001.zip › vetsci-4137191-supplementary.pdf]

Betulinic acid attenuates lipopolysaccharide-induced kidney inflammatory injury by suppressing PANoptosis in weaned piglets

Yu Yang[a,1], Huan Yao[a,1], Jiayu He[1], Zhaoping Ou[1], You Huang[1], Wenyu Ba[1], Ziming Wang[1], Jiao Wu[1], Hongyi Ding[1], Zhuliang Tan[1,2], Quanwei Li[1], Jine Yi[1,*], Shuiping Liu[1,*]

[1] Hunan Engineering Research Center of Livestock and Poultry Health Care, College of Veterinary Medicine, Hunan Agricultural University, Changsha, 410128, China

[2] SuBait Inc, Dartmouth, NS, B2W 6K4, Canada

[a] The authors contributed equally to this work

* Corresponding authors:

Jine Yi, College of Veterinary Medicine, Hunan Agricultural University, Changsha, Hunan Province, 410128, China. E-mail: yijine@hunau.edu.cn;

Shuiping Liu, College of Veterinary Medicine, Hunan Agricultural University, Changsha, Hunan Province, 410128, China. E-mail: liushuiping@hunau.edu.cn.

Table S1. Ingredients and nutrition levels of basal diet for the piglets (as-fed basis, %).

Item	Content, %
**Ingredients**	
Corn	61.3
Rice husk powder	2.5
Bean oil	1
Bean pulp	17.5
Braised soybean	5
Whey powder	5
Chocolate milk powder	2.5
Imported fish	2
Vitamin-mineral premix[1]	0.13
Calcium perphosphate	1.2
Glucose oxidase	0.025
proteinase	0.025
Sodium chloride	0.5
Stone powder	0.5
L-Lysine HCl	0.6
DL-Methionine	0.08
L-Threonine	0.1
L-Tryptophan	0.04
Total	100
**Nutrient levels**	
Digestible energy, MJ/kg	14.48
Crude protein	19.65
Calcium	0.69
Available phosphorus	0.48
Lysine	1.39
Methionine	0.48

Threonine	0.75
Tryptophan	0.24

[1]The vitamin-mineral premix provided per kilogram of feed: vitamin A, 10,500 IU; vitamin D_3, 3000 IU; vitamin E, 30 IU; vitamin K_3, 3 mg; vitamin B_1, 2.5 mg; vitamin B_2, 7.5 mg; vitamin B_6, 3 mg; vitamin B_{12}, 0.03 mg; niacin, 30 mg; pantothenate, 15 mg; choline chloride, 400 mg; folic acid, 1.5 mg; biotin, 0.2 mg; Cu, 12.5 mg; Fe, 115 mg; Zn, 70 mg; Mn, 27.5 mg; I, 0.5 mg; Se, 0.35mg.

Table S2. Primer sequences used in real-time PCR.

Target	GeneBank number	Primer sequence	Size, bp
NGAL	NM_001244410.1	F: 5'TTTGTCCCAAGTCTCCAGCC3'<br>R: 5'GTCGATGCACTGGTCGATTG3'	285
TNF-α	NM_397086	F: 5'GCCCAAGGACTCAGATCATCG3'<br>R: 5'TGTCTTTCAGCTTCACGCCGTTG3'	142
IL-1β	XM_397122	F: 5'GAAGCCGATGAAGAATCCCTC3'<br>R: 5'CAAAGTCATCATTGCACGTTT3'	116
IL-6	NM_214399.1	F: 5'CATTAAGTACATCCTCGGCAA3'<br>R: 5'TGTTTTCTGCCAGTACCTCC3'	98
IL-10	NM_397106	F: 5'TTCCATTCCAAGCCTACCCAC3'<br>R: 5'GCCACCGGAATATTAGCTGT3'	165
FAS	NM_213839.1	F: 5'ACACCAACCAGCAACACCAAATG3'<br>R: 5'CCAGTGCAGGTACGGGAATGAG3'	109
Caspase3	NM_214131.1	F: 5'GCTGTAGAACTCTAACTGGCAAACCC3'<br>R: 5'AGTCCCACTGTCCGTCTCAATCC3'	95
TNFR1	XM_021068189.1	F: 5'CCTTTCCGGAACAAGGTCAC3'<br>R: 5'CGTTTGTCTCCACGATGCAC3'	229
RIPK1	XM_005665537.3	F: 5'CCAAGCGCAATGAGTACAAC3'<br>R: 5'CCCTTCACAGACAGAGGGAC3'	196
RIPK3	XM_001927424.4	F: 5'GGCGTTAAGTTATGGCACGTT3'<br>R: 5'CGTGCCAAATCCTCCTTTGC3'	96
MLKL	XM_003481791.4	F: 5'GCTTCAGGTGGATCAACGGATG3'<br>R: 5'GTCCTTGGATAACTCGCTTGTCTTC3'	107
Caspase1	NM_214162.1	F: 5'ATCTCACCGCTTCGGACAT3'<br>R: 5'GCCAGCCTGGATTCCATGAG3'	113
NLRP3	NM_001256770.2	F: 5'GCAAGCTGGCTCGTTACCTG3'<br>R: 5'ACAGAAAGATTTGCATTGTCCCAT3'	280
PYCARD<br>*（ASC）*	XM_003124468.5	F: 5'TGCGTGACATCGGCATGAAG3'<br>R: 5'CACGAAGTGCAGTGCTGGTTTG3'	131
HMGB1	NM_001004034.1	F: 5'CTCAGAAAGGTGGAAGACCATGT3'<br>R: 5'TGGGTGCATTGGGATCCTTG3'	152
TLR4	NM_001113039.2	F: 5'ATGATTCCTCGCATCCGCCT3'<br>R: 5'GTTAGGAACCACCTGCACGC3'	105
NF-κB	NM_001114281.1	F: 5'CATCTTTGACAACCGTGCCC3'<br>R: 5'CTTTCTGCACCTTGTCGCAC3'	119
IKK-β	XM_021077168.1	F: 5'CTGCCTGTCCAAGATGAAGAACTCC3'<br>R: 5'GTCCGATGTGATCCCAAACTCTGTC3'	136
GAPDH	NM_001289726.2	F: 5'GCTGCCCAGAACATCATCCC3'<br>R: 5'GATGTCATCATACTTGGCAGGTT3'	171

Table S3. The primary protein antibodies used in Western blot analysis.

Name of antibody	Company of production
Bax	Abmart, Shanghai, China
Bcl-2	Abmart, Shanghai, China
Cleaved caspase 3	Wanleibio, Shenyang, China
RIPK1	Wanleibio, Shenyang, China
p-RIPK3	Zenbio, Chengdu, China
RIPK3	ABclonal, Wuhan, China
p-MLKL	Zenbio, Chengdu, China
MLKL	ABclonal, Wuhan, China
NLRP3	Wanleibio, Shenyang, China
ASC	Wanleibio, Shenyang, China
Cleaved caspase 1	Wanleibio, Shenyang, China
IL-1β	Wanleibio, Shenyang, China
GSDMD-N	Wanleibio, Shenyang, China
HMGB1	Zenbio, Chengdu, China
TLR4	Bioss, Beijing, China
p-P65	Abmart, Shanghai, China
P65	Abmart, Shanghai, China
β-actin	ABclonal, Wuhan, China
