# Supplementary figures and images for: Betulinic Acid Attenuates Lipopolysaccharide-Induced Kidney Inflammatory Injury by Suppressing PANoptosis in Weaned Piglets
Source: Vet Sci. 2026 Feb 25;13(3):213. doi: 10.3390/vetsci13030213 (PMC13030141; doi:10.3390/vetsci13030213)

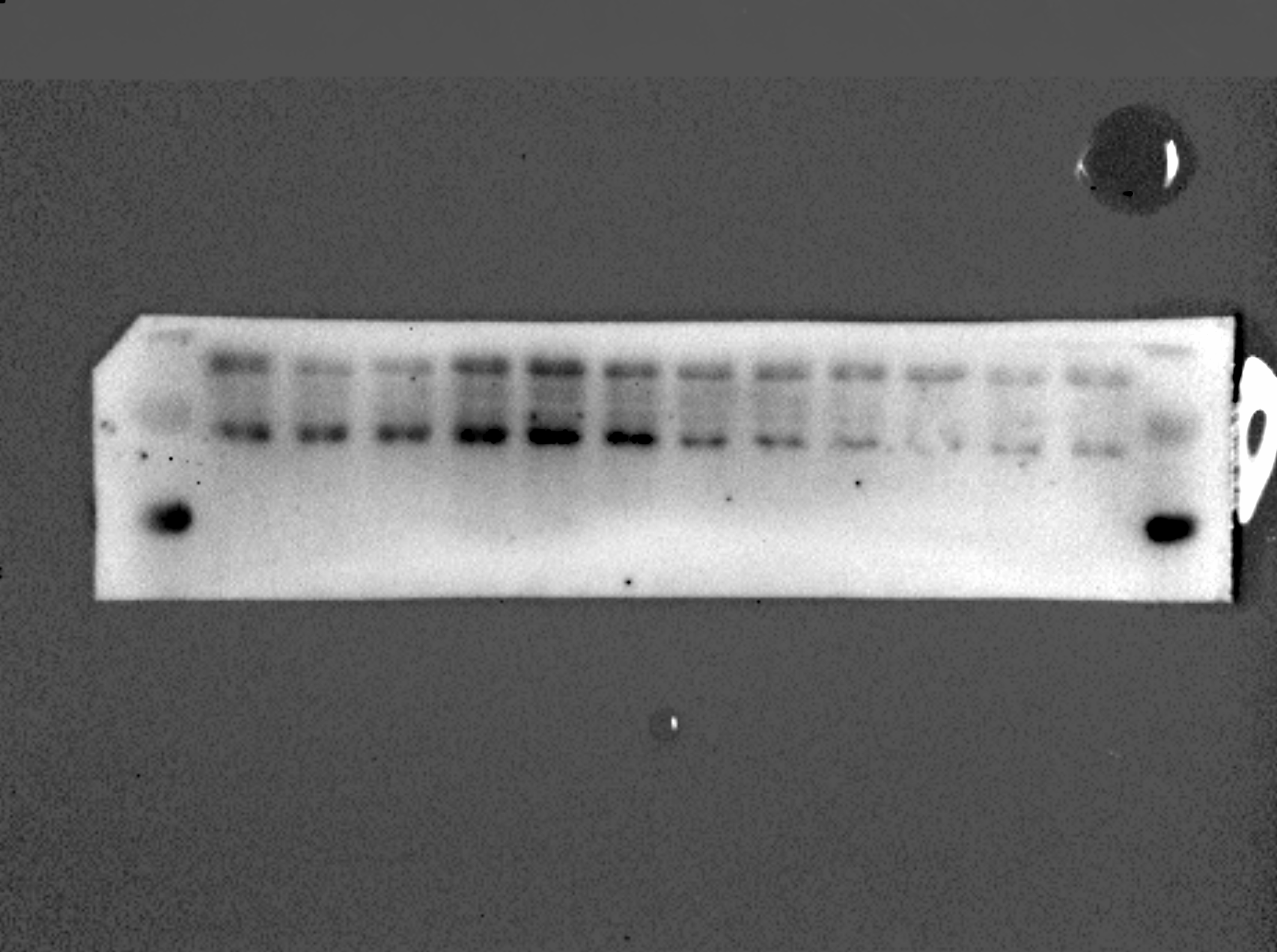

Supplement: Supplementary file 1 [file vetsci-13-00213-s001.zip › WB Original image/ASC.tif]

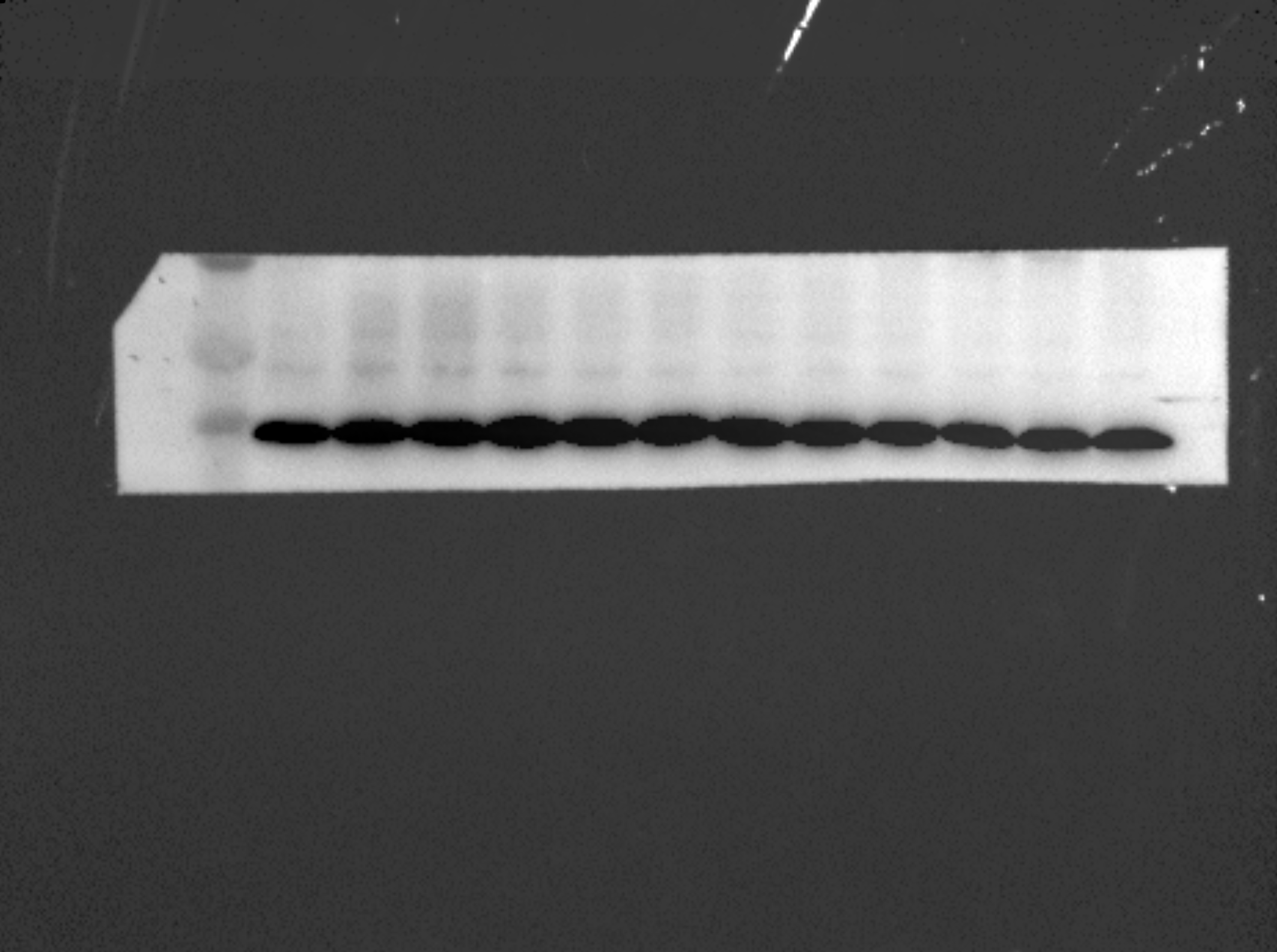

Supplement: Supplementary file 1 [file vetsci-13-00213-s001.zip › WB Original image/Bax.tif]

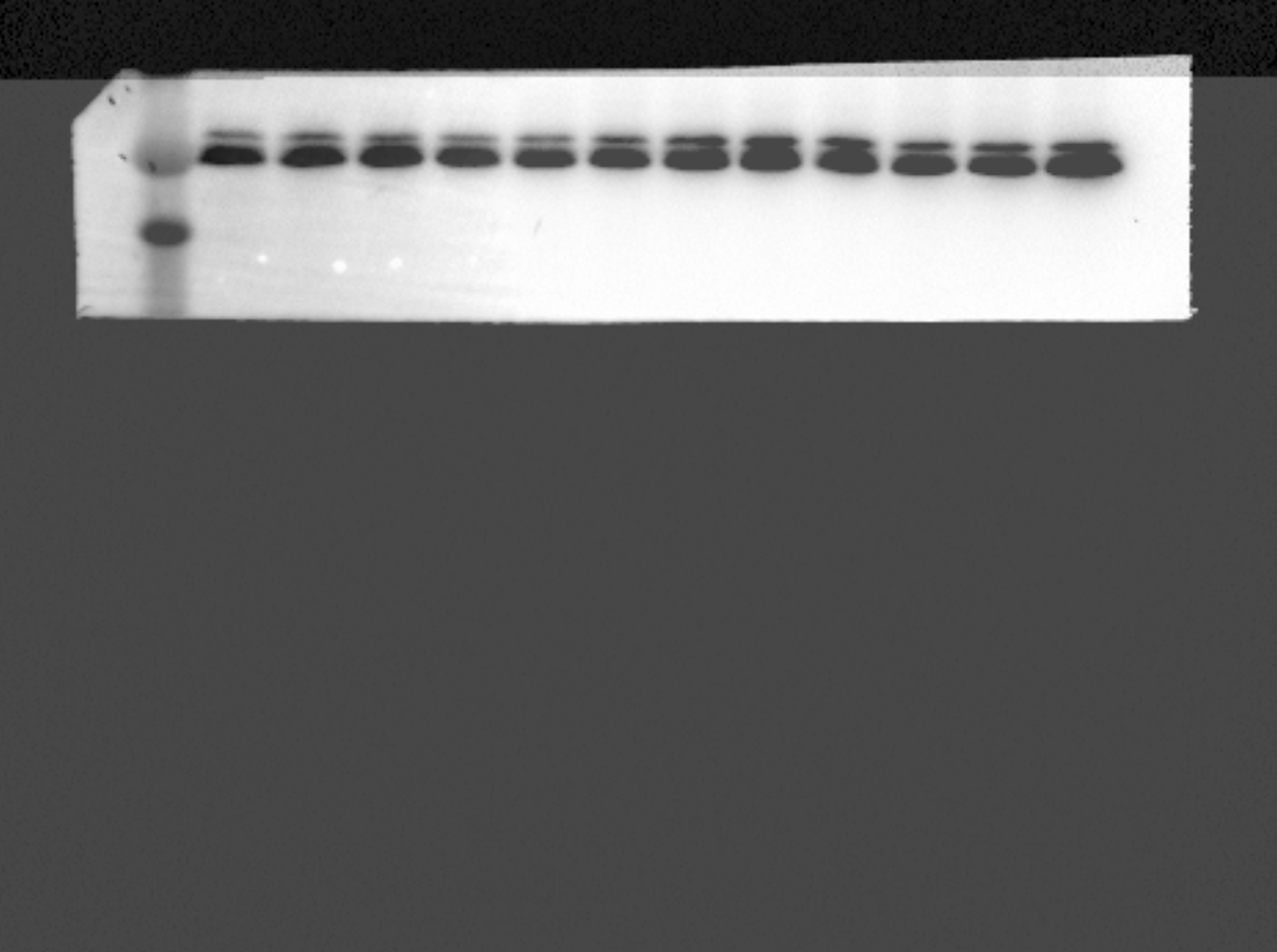

Supplement: Supplementary file 1 [file vetsci-13-00213-s001.zip › WB Original image/Bcl-2.tif]

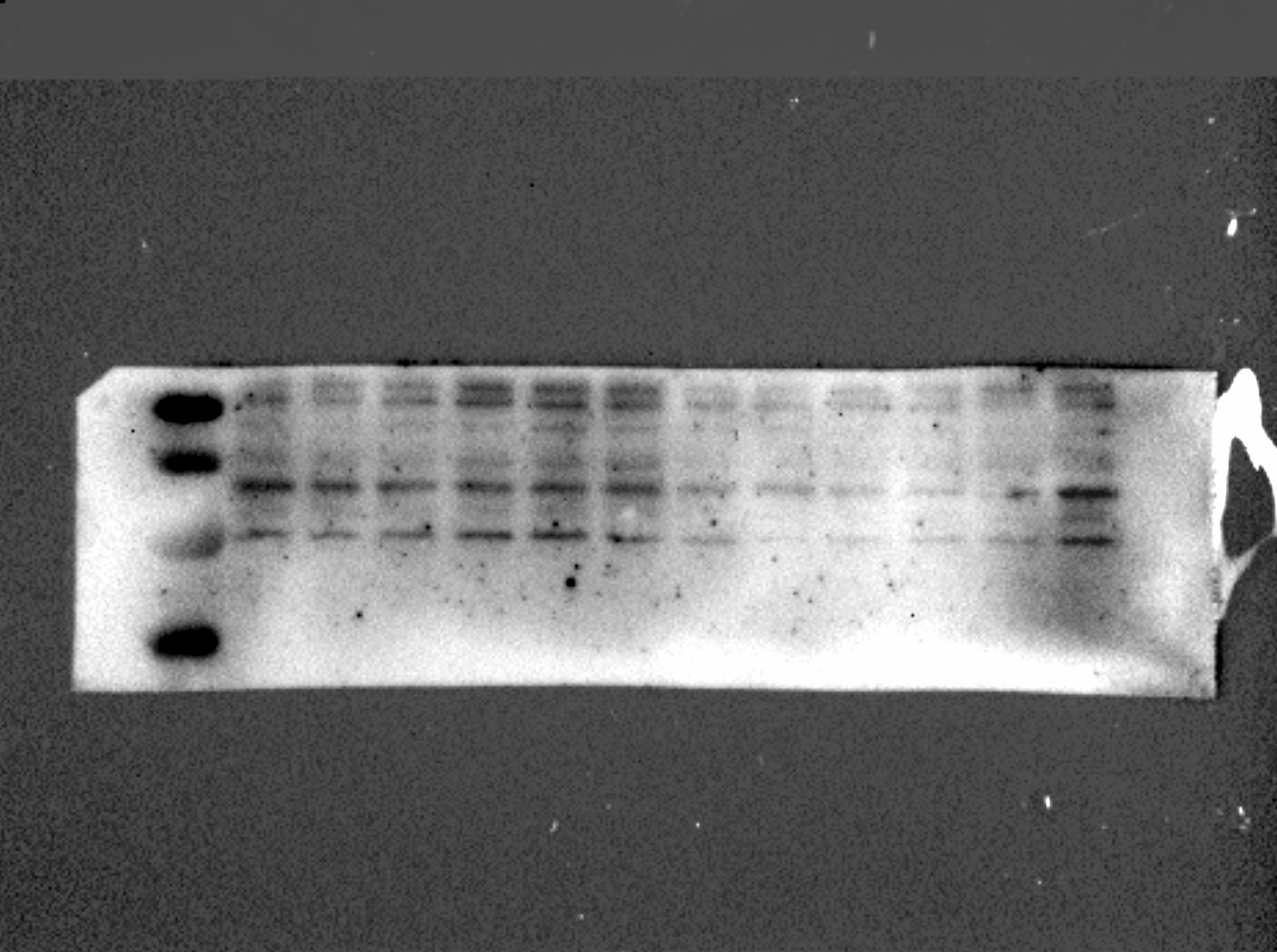

Supplement: Supplementary file 1 [file vetsci-13-00213-s001.zip › WB Original image/cleaved Caspase-1.tif]

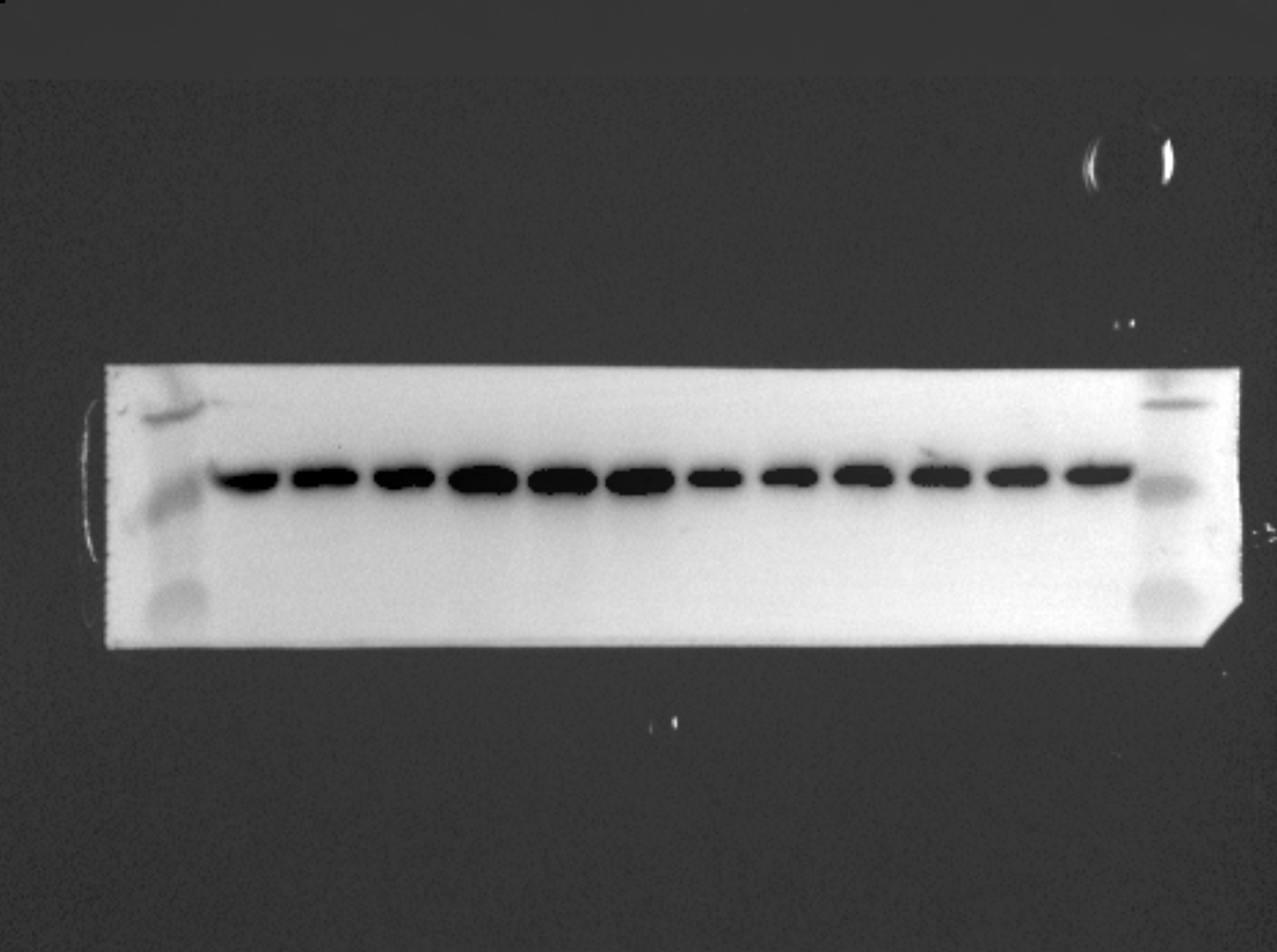

Supplement: Supplementary file 1 [file vetsci-13-00213-s001.zip › WB Original image/cleaved Caspase-3.tif]

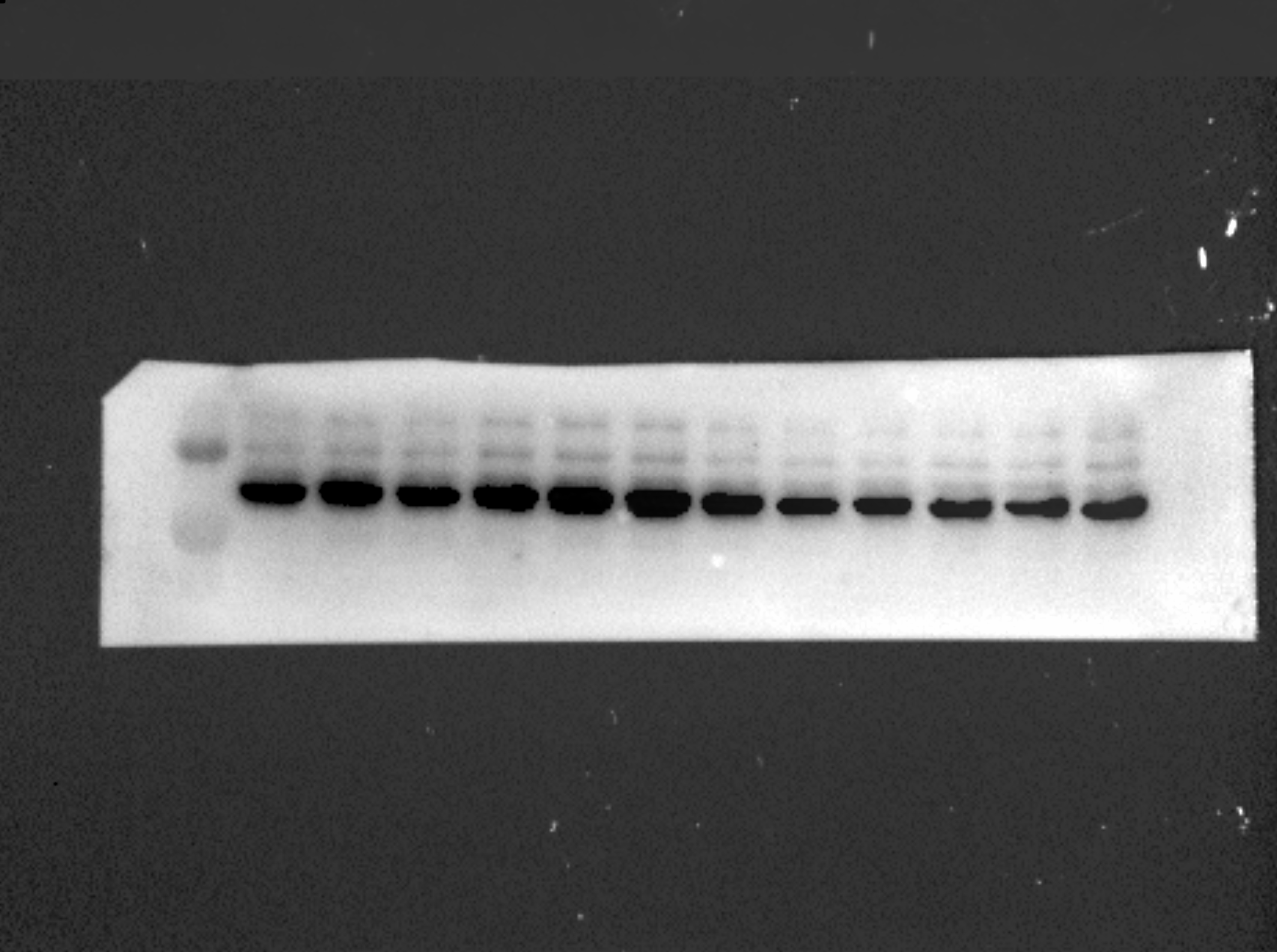

Supplement: Supplementary file 1 [file vetsci-13-00213-s001.zip › WB Original image/GSDMD.tif]

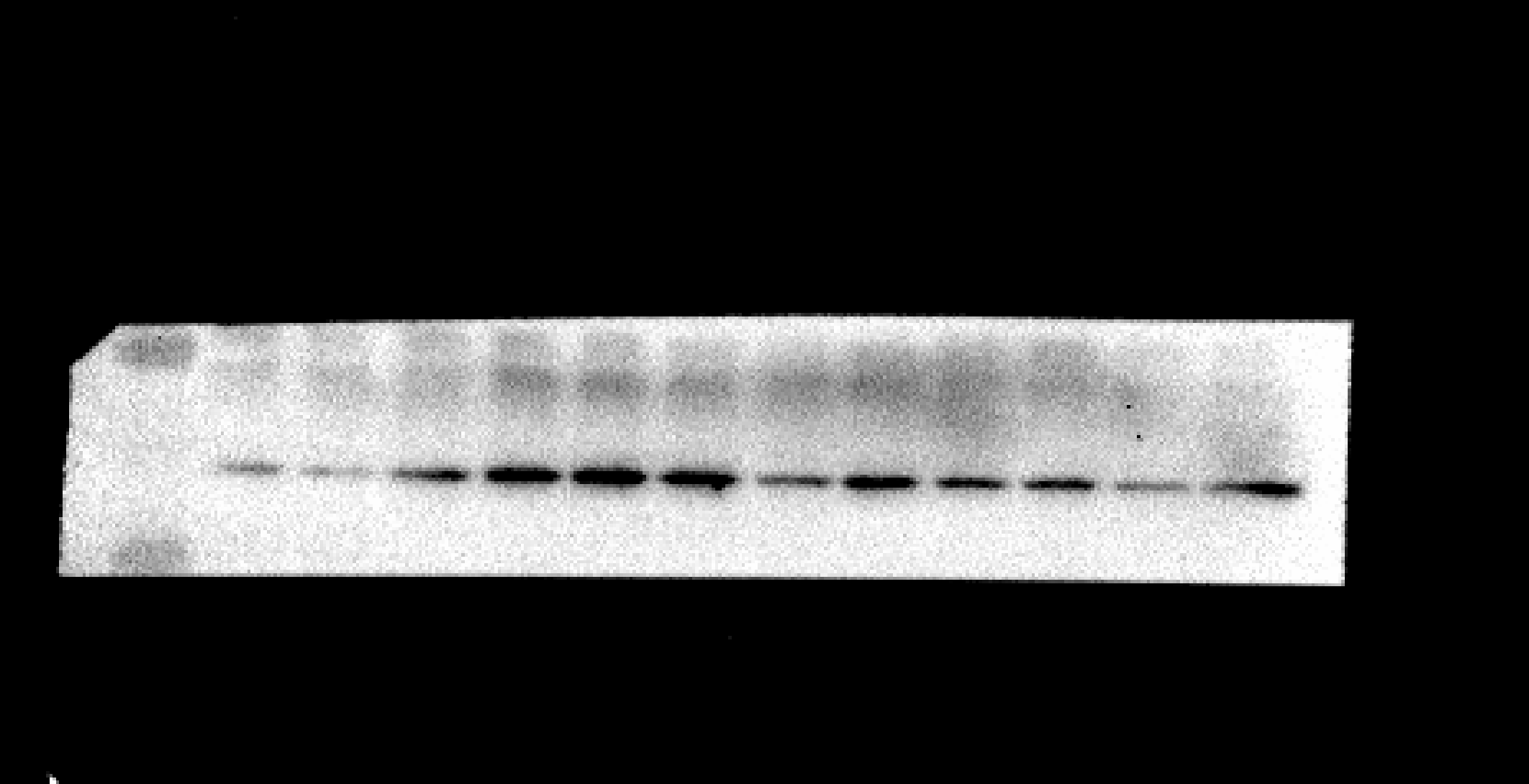

Supplement: Supplementary file 1 [file vetsci-13-00213-s001.zip › WB Original image/HMGB1.tif]

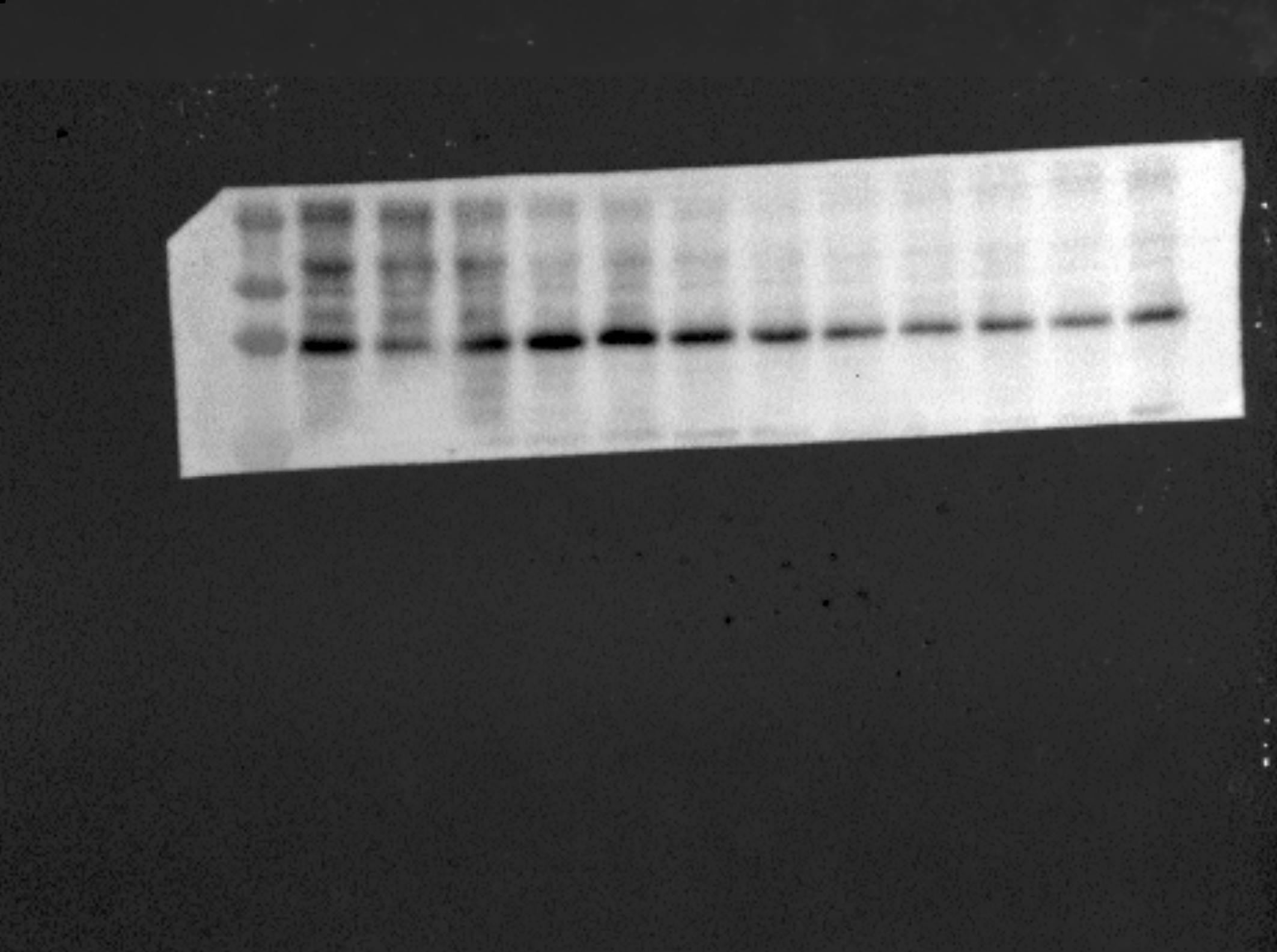

Supplement: Supplementary file 1 [file vetsci-13-00213-s001.zip › WB Original image/IL-1β.tif]

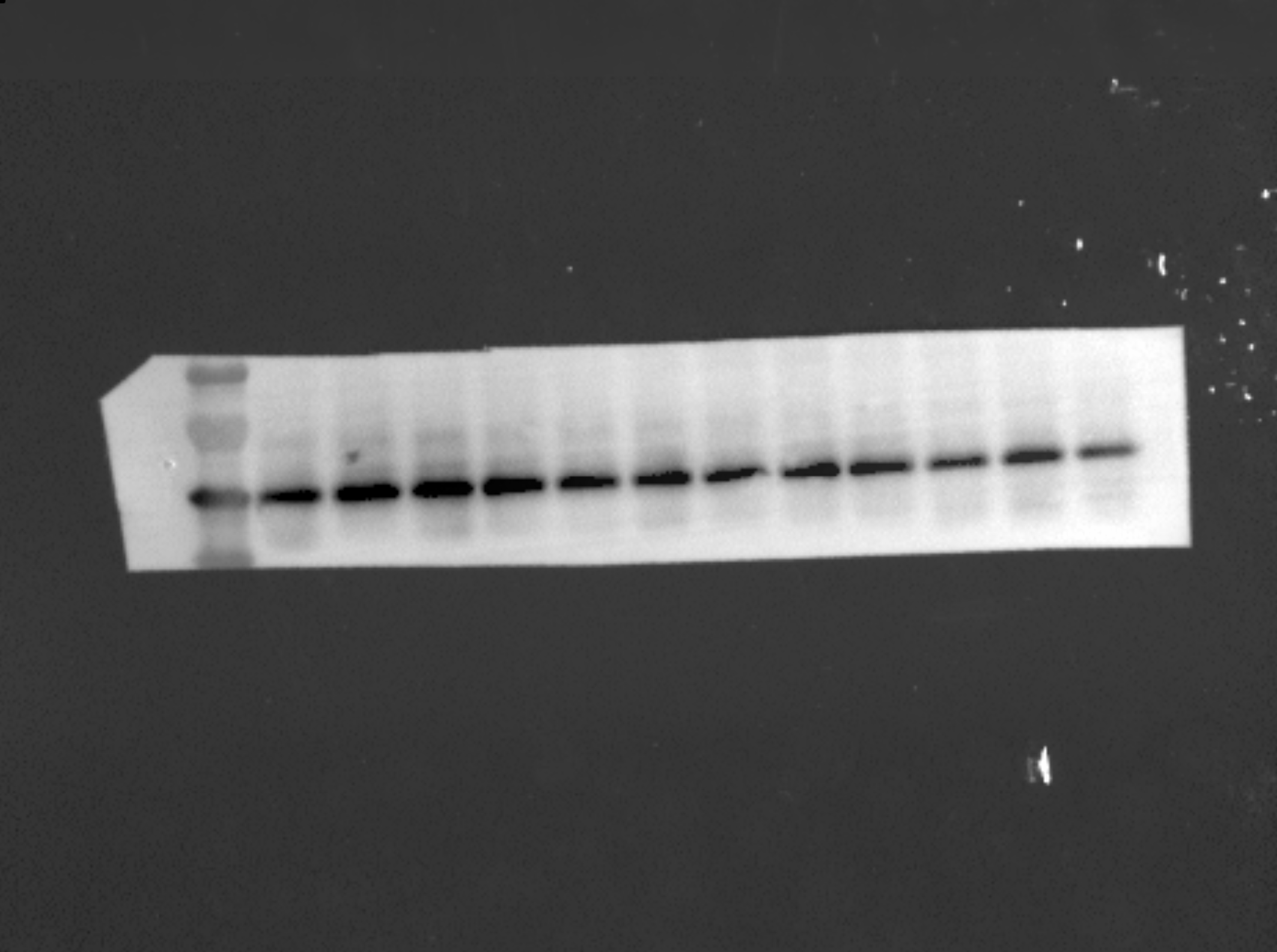

Supplement: Supplementary file 1 [file vetsci-13-00213-s001.zip › WB Original image/MLKL.tif]

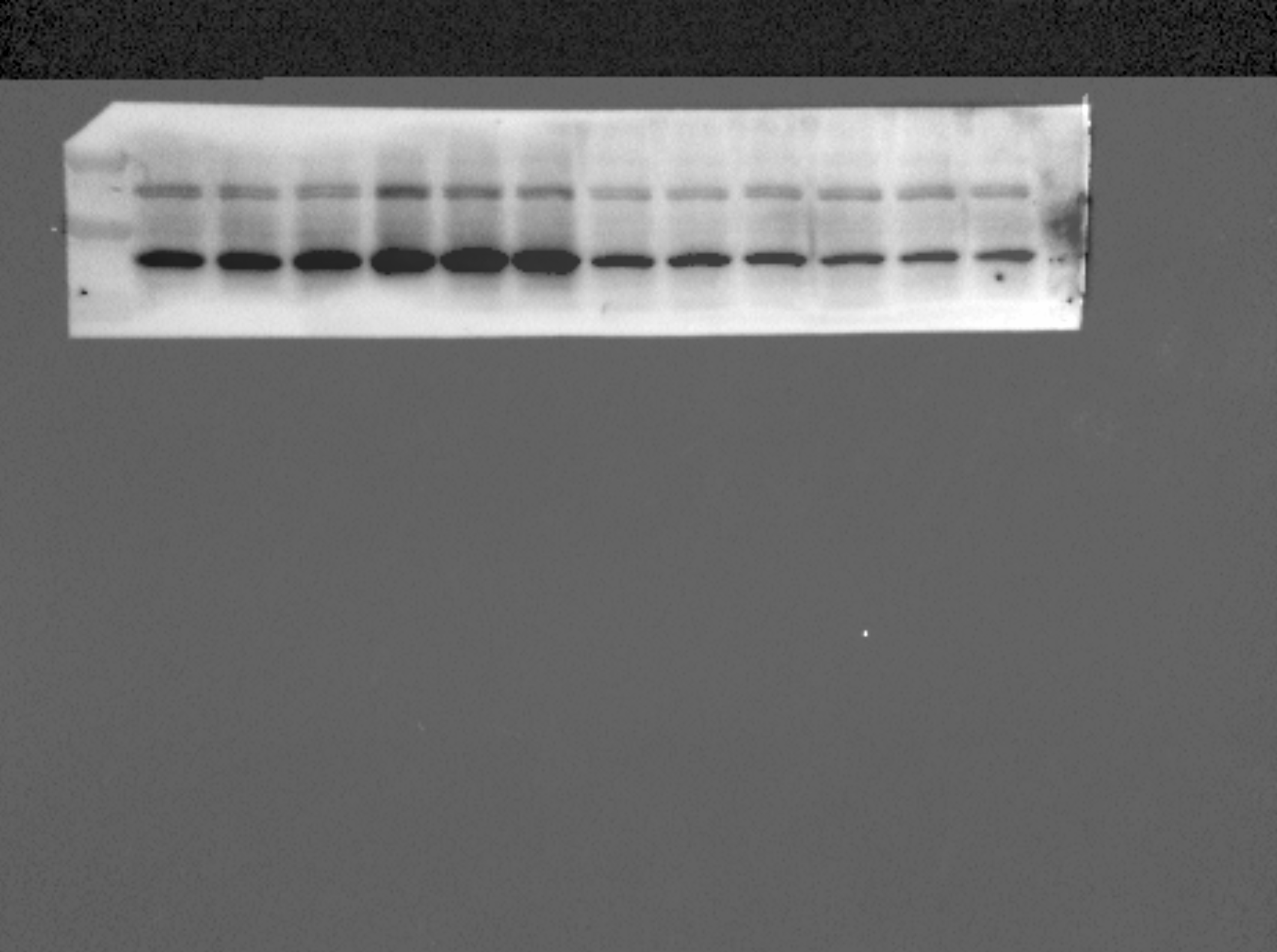

Supplement: Supplementary file 1 [file vetsci-13-00213-s001.zip › WB Original image/NLRP3.tif]

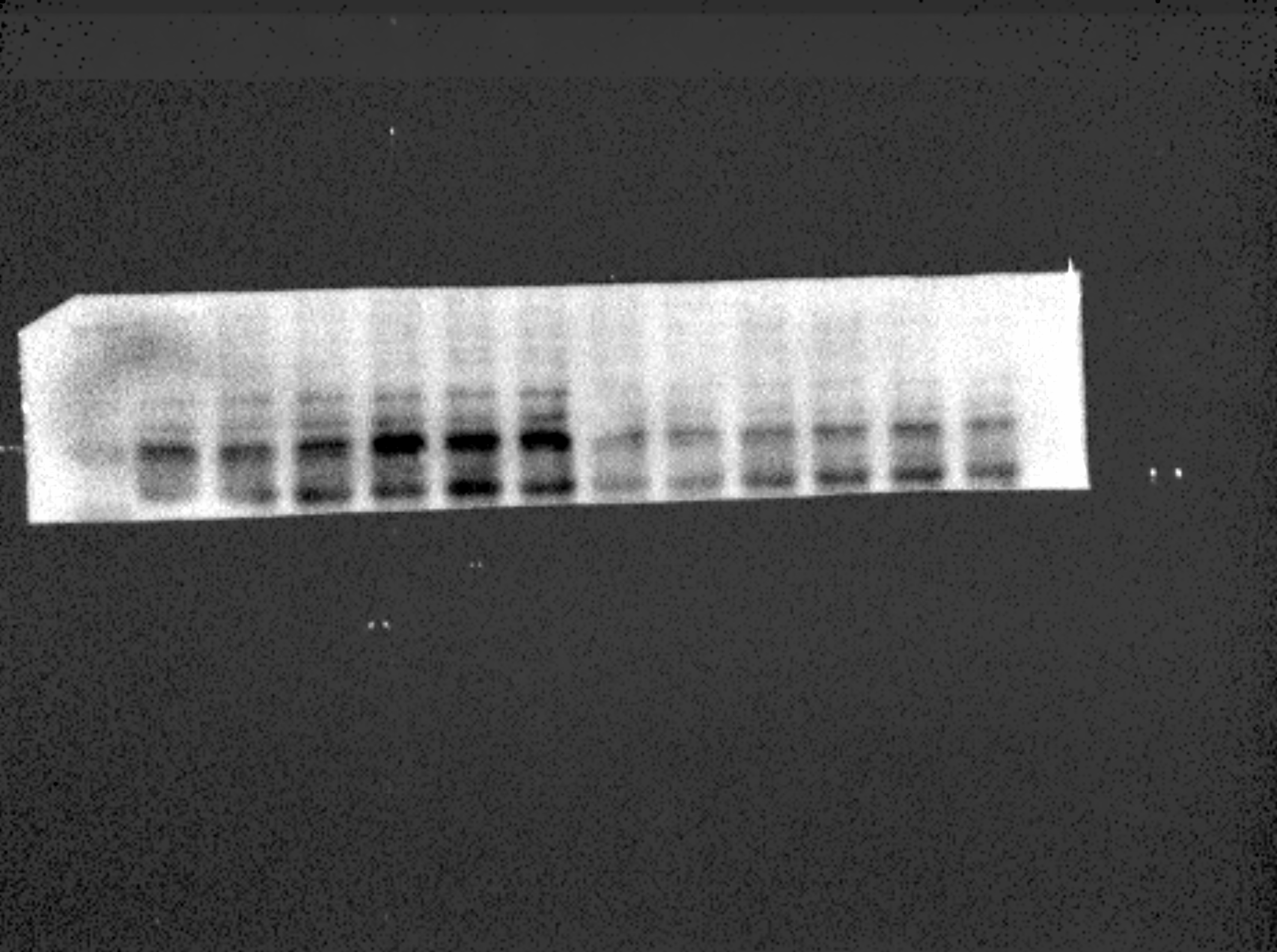

Supplement: Supplementary file 1 [file vetsci-13-00213-s001.zip › WB Original image/p-MLKL.tif]

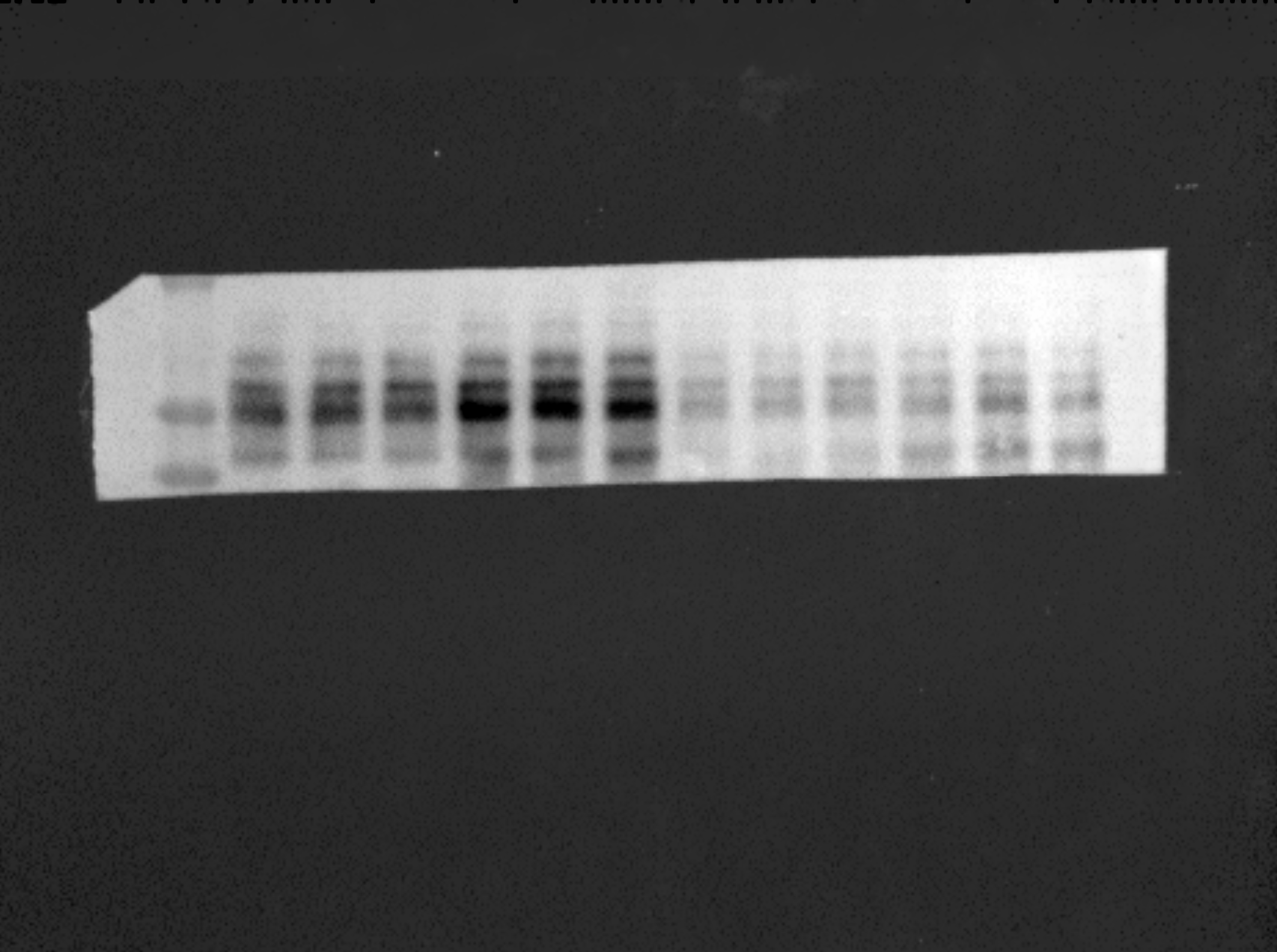

Supplement: Supplementary file 1 [file vetsci-13-00213-s001.zip › WB Original image/p-p65.tif]

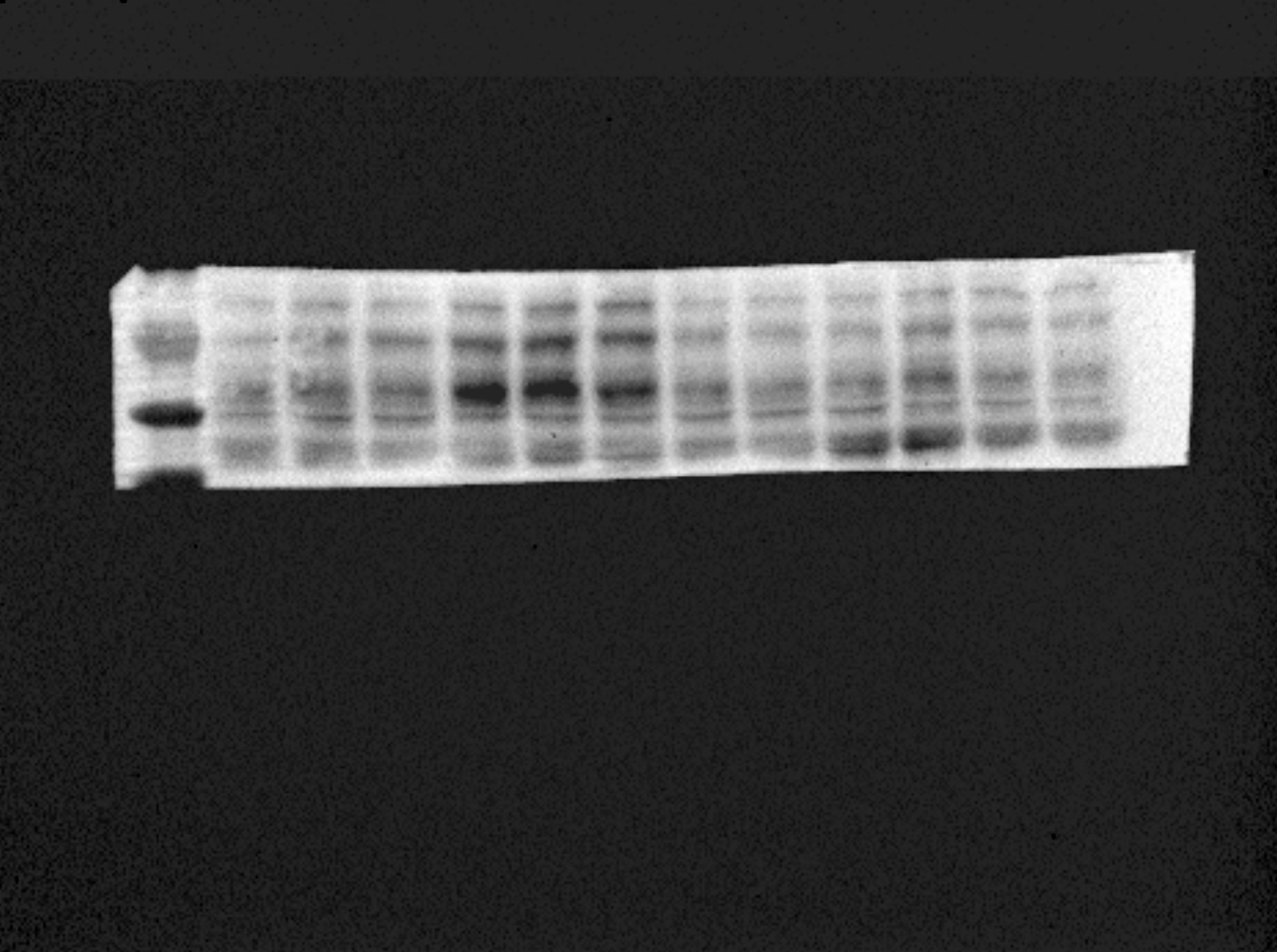

Supplement: Supplementary file 1 [file vetsci-13-00213-s001.zip › WB Original image/p-RIPK3.tif]

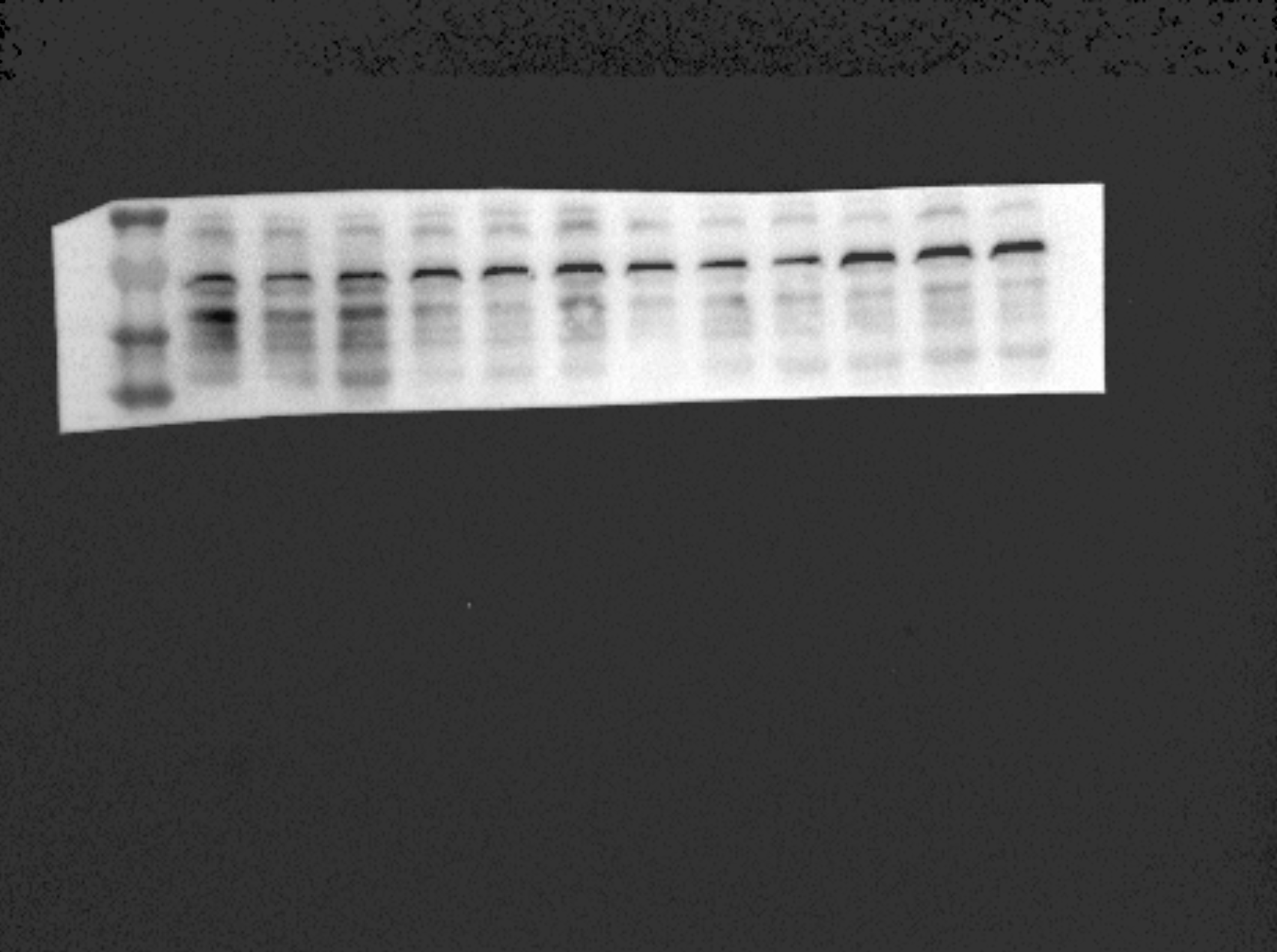

Supplement: Supplementary file 1 [file vetsci-13-00213-s001.zip › WB Original image/p65.tif]

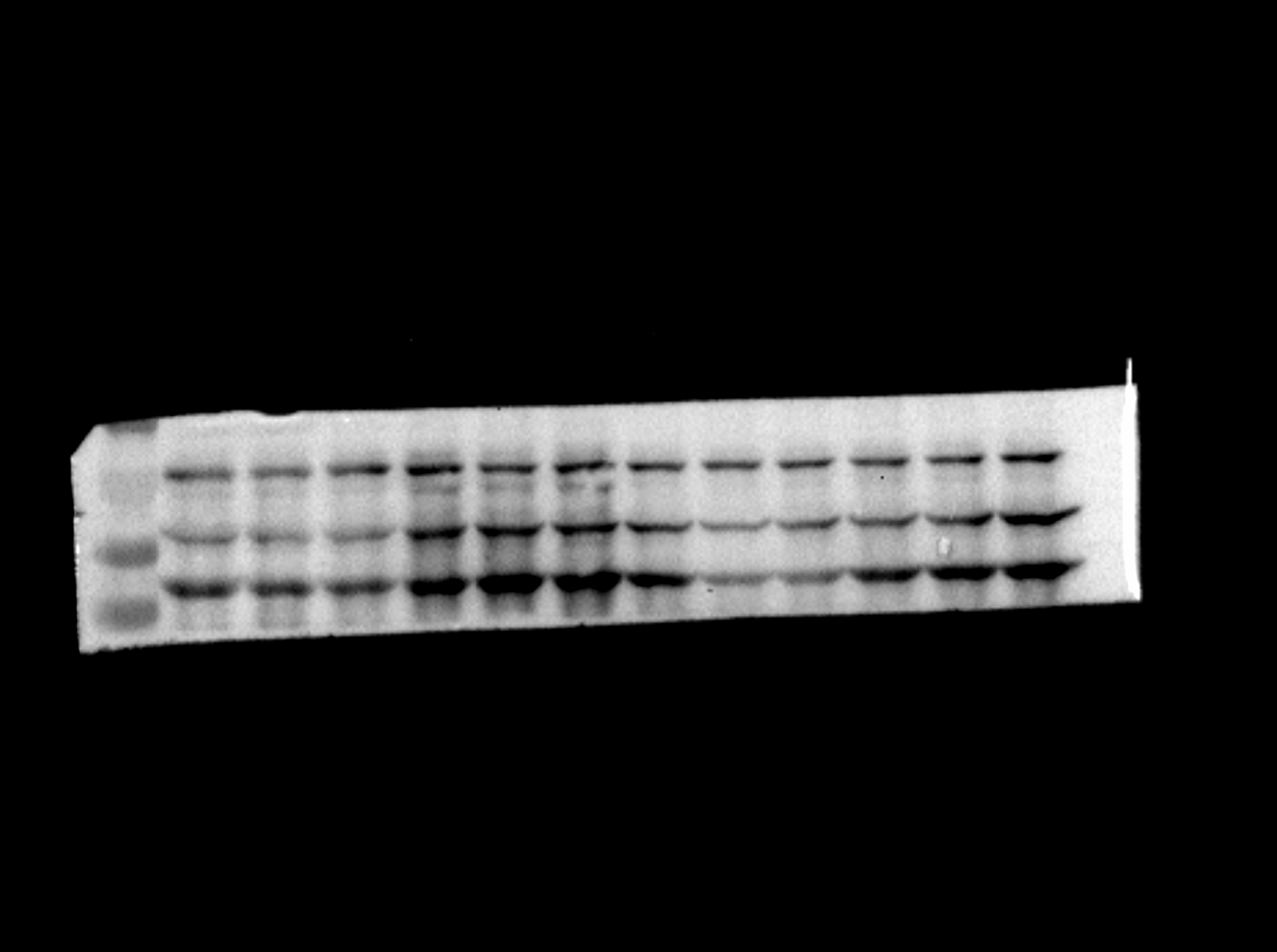

Supplement: Supplementary file 1 [file vetsci-13-00213-s001.zip › WB Original image/RIPK1.tif]

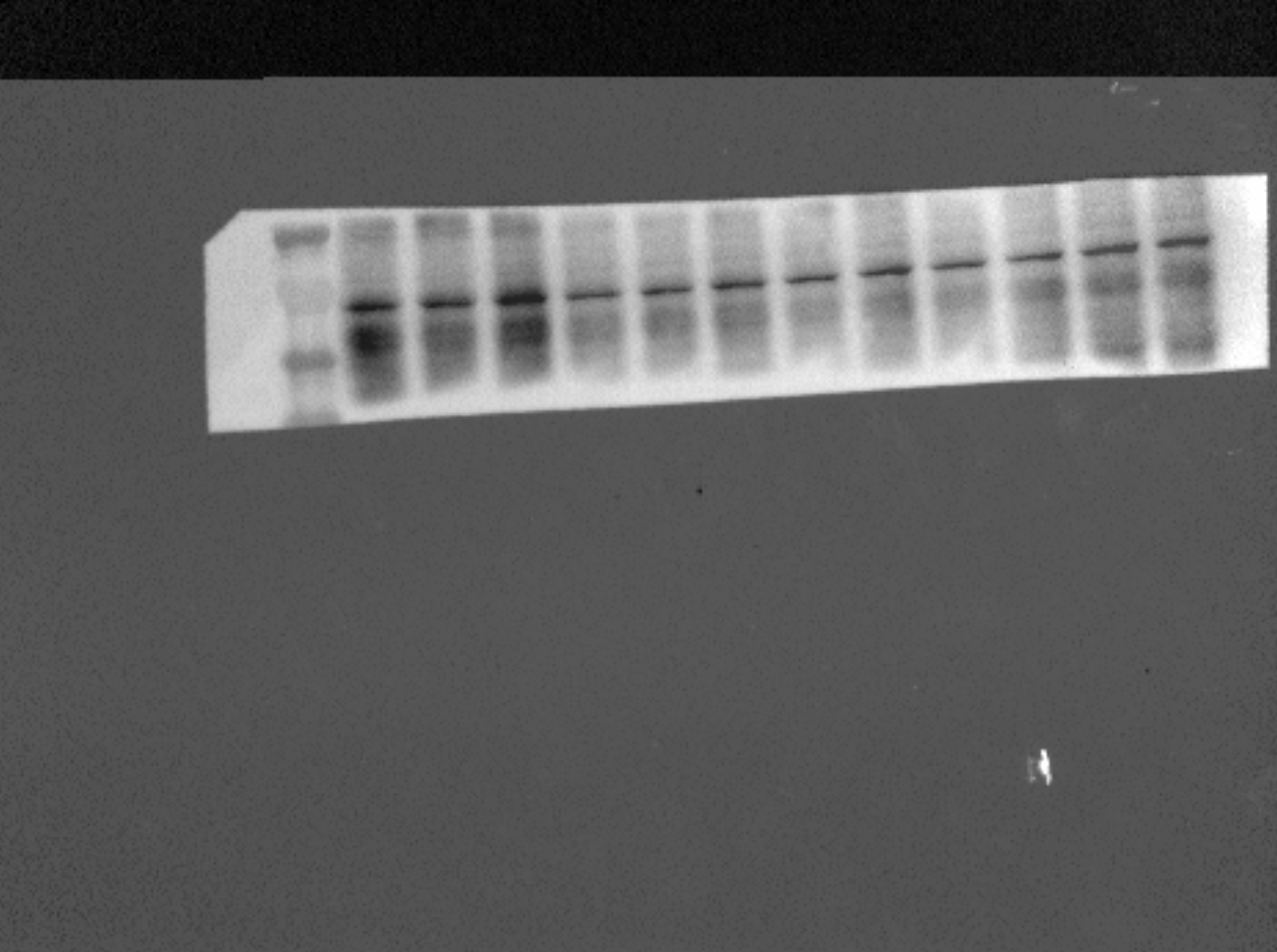

Supplement: Supplementary file 1 [file vetsci-13-00213-s001.zip › WB Original image/RIPK3.tif]

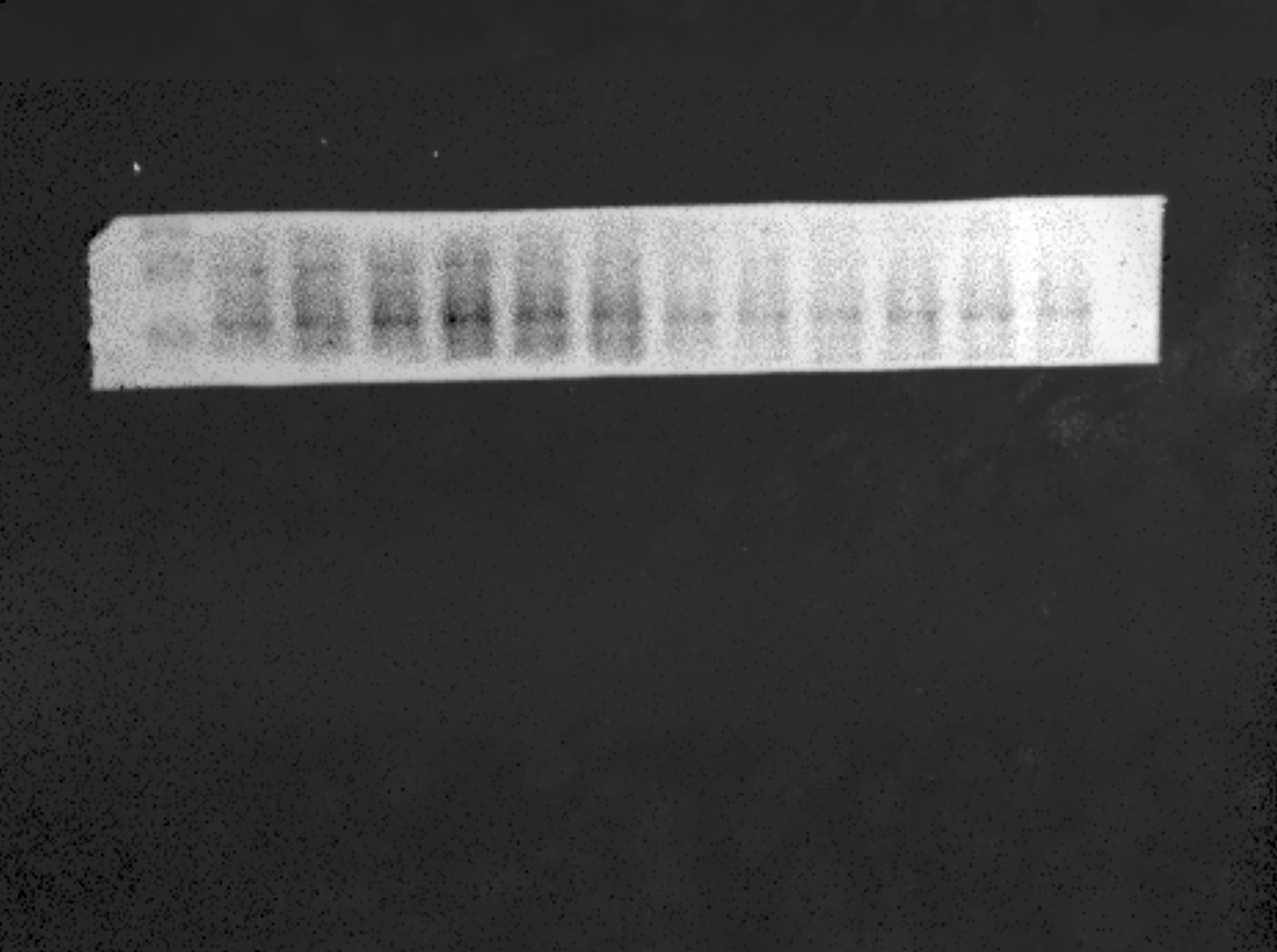

Supplement: Supplementary file 1 [file vetsci-13-00213-s001.zip › WB Original image/TLR4.tif]

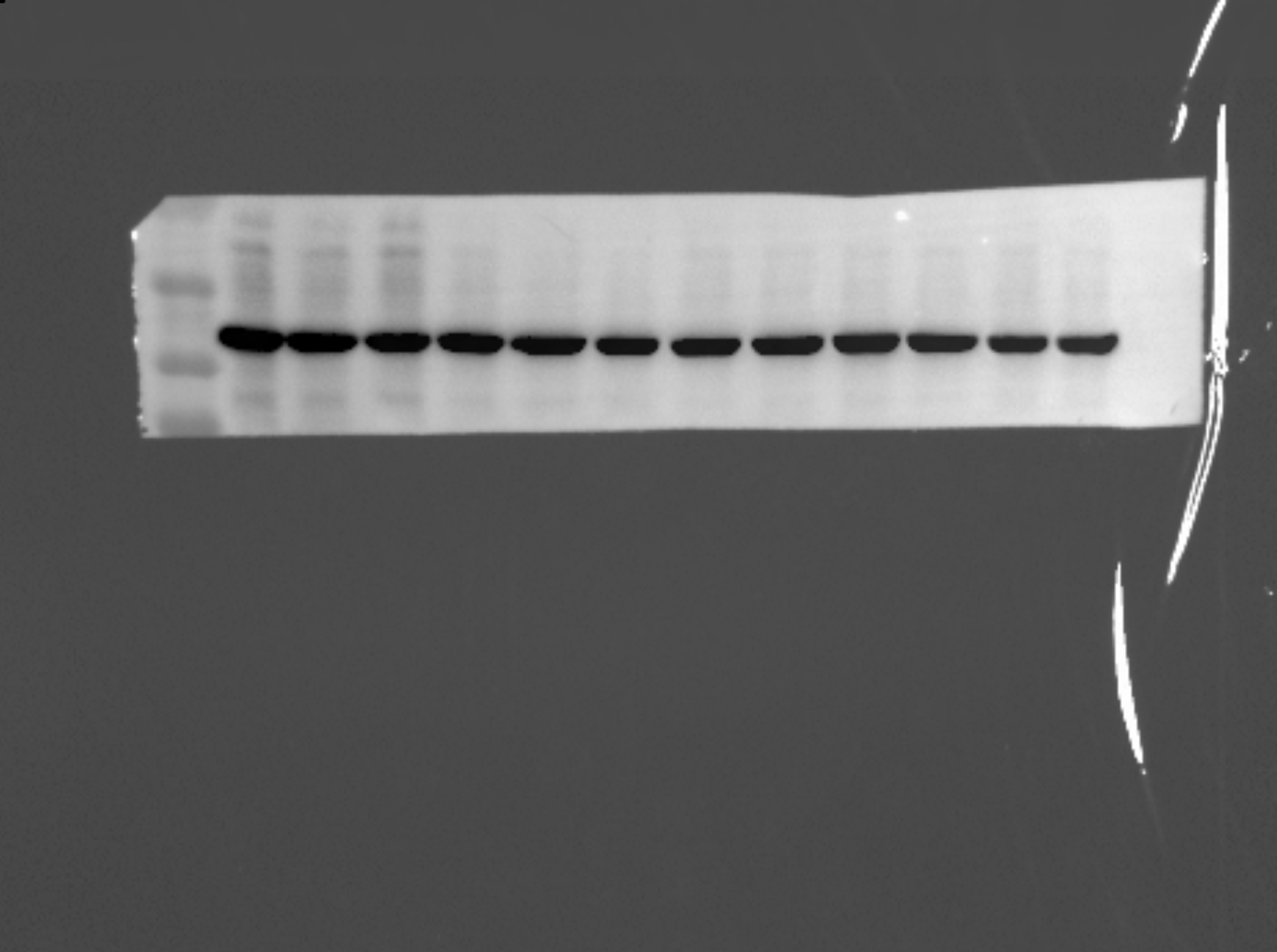

Supplement: Supplementary file 1 [file vetsci-13-00213-s001.zip › WB Original image/凋亡actin.tif]

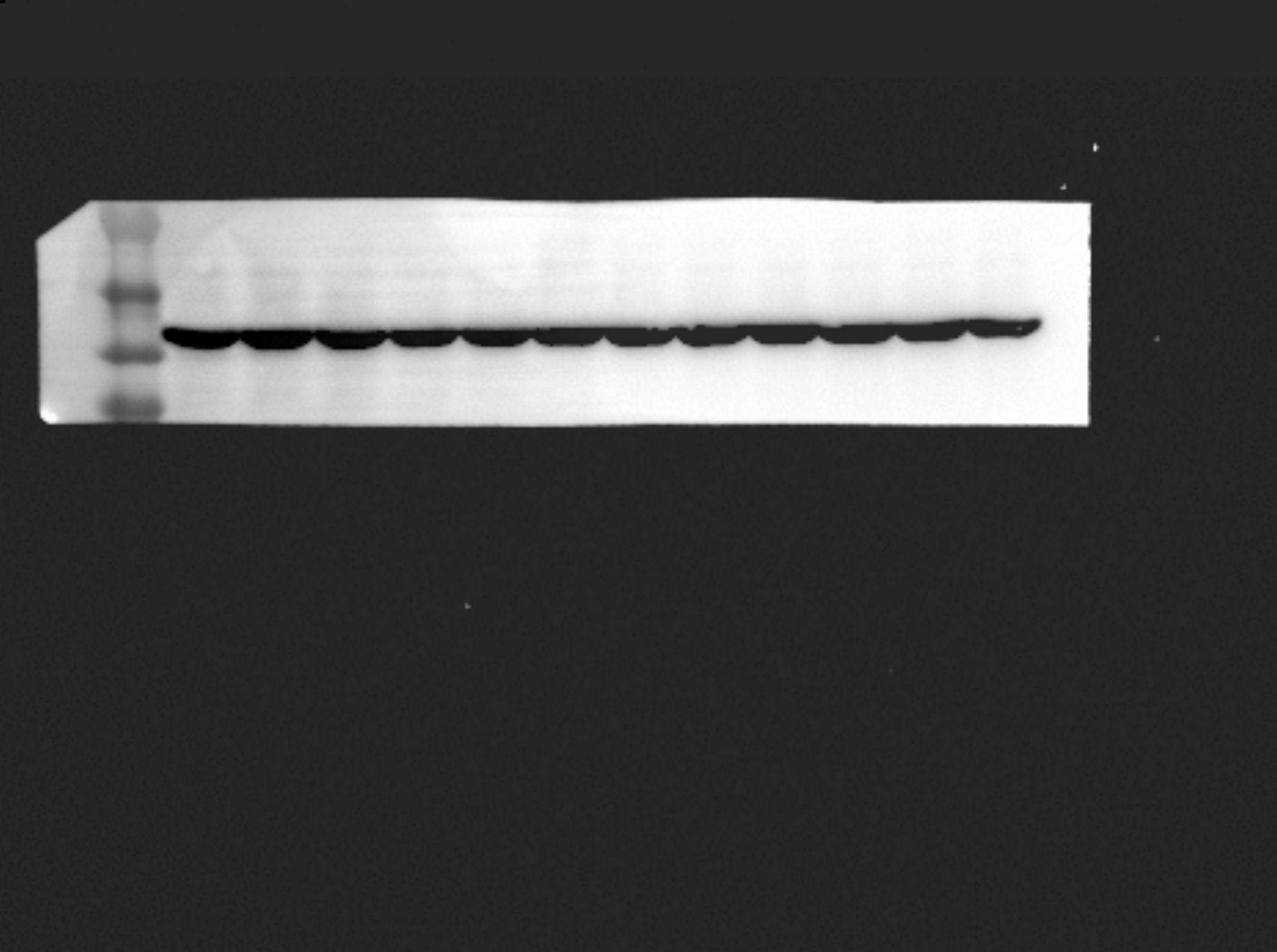

Supplement: Supplementary file 1 [file vetsci-13-00213-s001.zip › WB Original image/坏死性凋亡actin.tif]

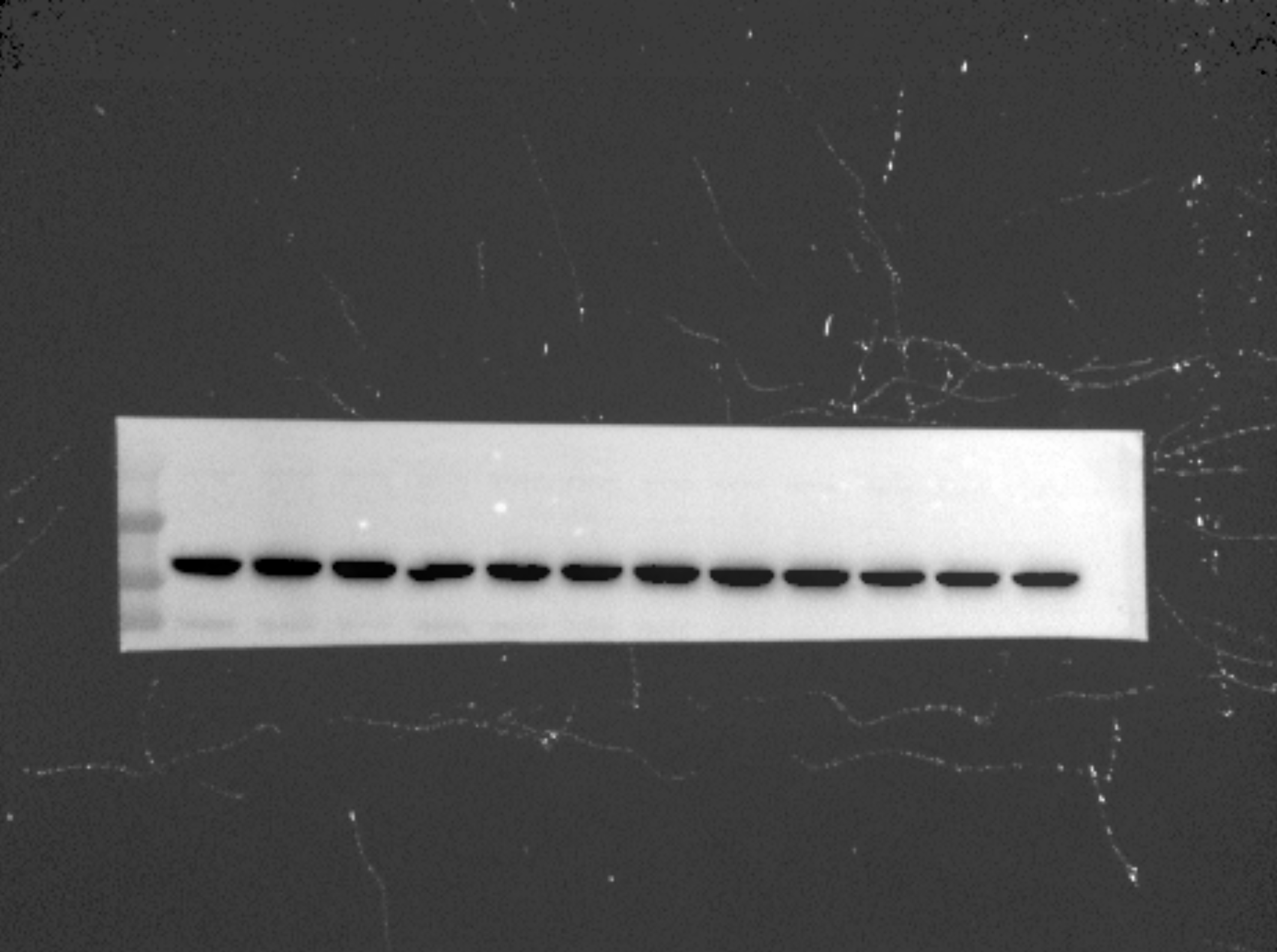

Supplement: Supplementary file 1 [file vetsci-13-00213-s001.zip › WB Original image/焦亡actin.tif]

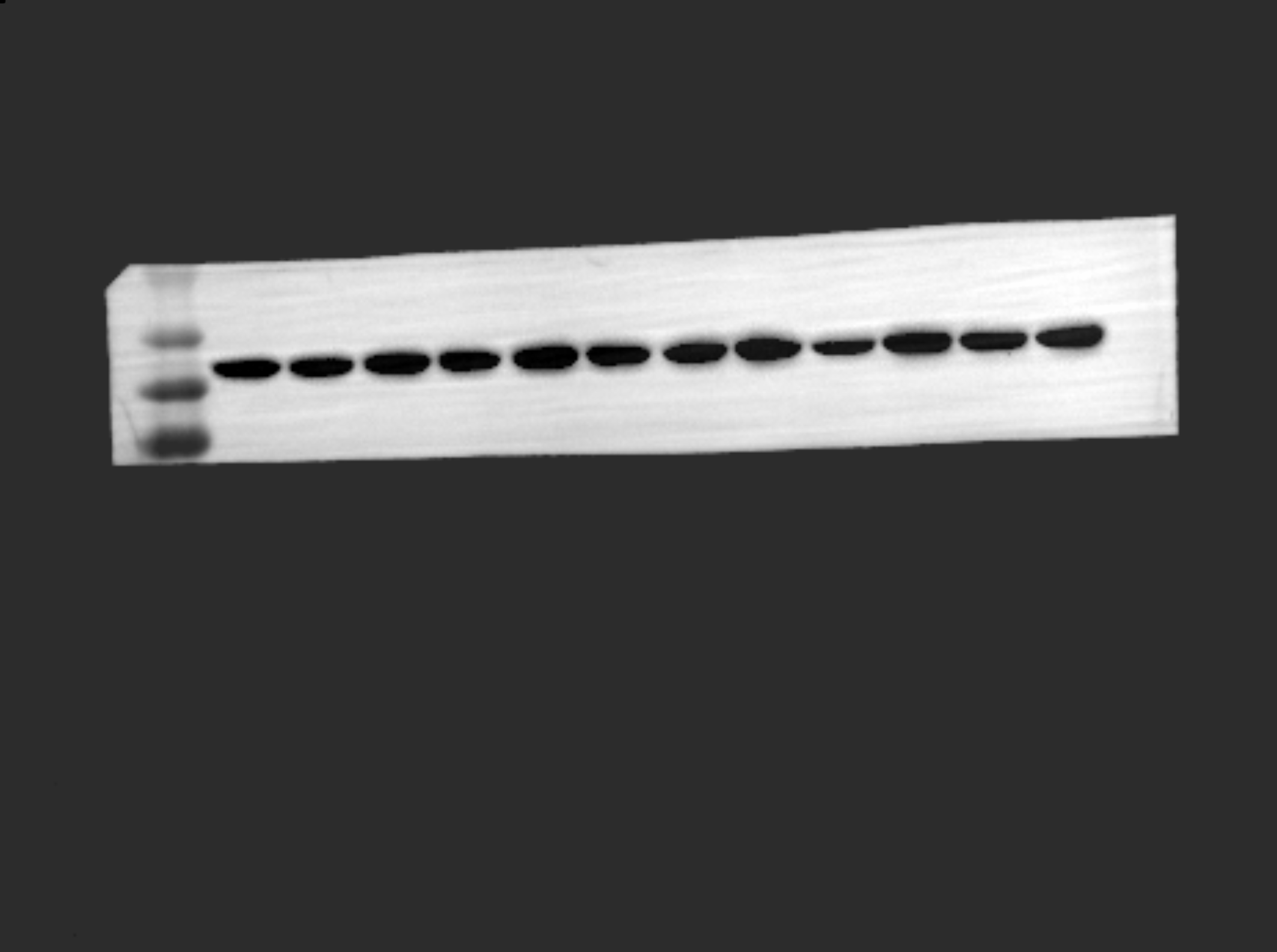

Supplement: Supplementary file 1 [file vetsci-13-00213-s001.zip › WB Original image/通路actin.tif]
